# Structural insights into the interaction and disease mechanism of neurodegenerative disease-associated optineurin and TBK1 proteins

**DOI:** 10.1038/ncomms12708

**Published:** 2016-09-13

**Authors:** Faxiang Li, Xingqiao Xie, Yingli Wang, Jianping Liu, Xiaofang Cheng, Yujiao Guo, Yukang Gong, Shichen Hu, Lifeng Pan

**Affiliations:** 1 State Key Laboratory of Bioorganic and Natural Products Chemistry, Shanghai Institute of Organic Chemistry, Chinese Academy of Sciences, 345 Lingling Road, Shanghai 200032, China; 2 Interdisciplinary Research Center on Biology and Chemistry, Shanghai Institute of Organic Chemistry, Chinese Academy of Sciences, 345 Lingling Road, Shanghai 200032, China; 3 Collaborative Innovation Center of Chemistry for Life Sciences, Shanghai Institute of Organic Chemistry, Chinese Academy of Sciences, 345 Lingling Road, Shanghai 200032, China

## Abstract

Optineurin is an important autophagy receptor involved in several selective autophagy processes, during which its function is regulated by TBK1. Mutations of optineurin and TBK1 are both associated with neurodegenerative diseases. However, the mechanistic basis underlying the specific interaction between optineurin and TBK1 is still elusive. Here we determine the crystal structures of optineurin/TBK1 complex and the related NAP1/TBK1 complex, uncovering the detailed molecular mechanism governing the optineurin and TBK1 interaction, and revealing a general binding mode between TBK1 and its associated adaptor proteins. In addition, we demonstrate that the glaucoma-associated optineurin E50K mutation not only enhances the interaction between optineurin and TBK1 but also alters the oligomeric state of optineurin, and the ALS-related TBK1 E696K mutation specifically disrupts the optineurin/TBK1 complex formation but has little effect on the NAP1/TBK1 complex. Thus, our study provides mechanistic insights into those currently known disease-causing optineurin and TBK1 mutations found in patients.

## Abstract (web summary)

Mutations in optineurin that cause defects in the interaction with TBK1 are associated with neurodegenerative diseases. Here, the authors report the structure of this complex, and outline a general binding mode for these proteins.

## Graphical abstract

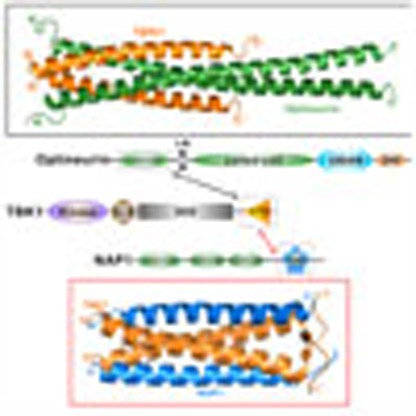

Autophagy is a well-conserved and highly regulated cellular degradative process in eukaryotic cells, during which undesired cytoplasmic materials, including damaged organelles, bulk protein aggregates and invasive pathogens are targeted for lysosome-dependent degradation^1^,^2^,^3^. Owing to its pivotal roles in cell's adaptation to various stresses and maintenance of cellular homeostasis, autophagy plays a critical role in numerous physiologic processes such as cell differentiation, immune response and ageing^2^,^4^,^5^. In contrast to non-selective ‘bulk' autophagy, selective autophagy relies on a panel of cargo adaptor proteins named autophagy receptors and the unique double-membraned vesicle termed autophagosome to selectively recognize and engulf cytosolic components for delivery to lysosome^6^,^7^,^8^. The currently known autophagy receptors in mammals, such as SQSTM1/p62, optineurin (OPTN), NBR1, CALCOCO2/NDP52, TAX1BP1, Nix, FUNDC1 and STBD1, all contain a cargo-associating domain, which can specifically recognize certain types of cytosolic cargoes, and a LC3-interacting region (LIR) motif that can recruit the ATG8 family proteins^7^,^8^. Thereby, autophagy receptors can function as bridging adaptors directly linking the specific cargoes to the autophagy machinery, and mediate the subsequent selective autophagy processes, such as aggrephagy (selective autophagy of bulk protein aggregates), xenophagy (selective autophagy of pathogens) and mitophagy (selective autophagy of mitochondria)^6^,^7^,^8^,^9^. Given the fundamental roles played by autophagy receptors in selective autophagy, the functions of autophagy receptors have been temporally and spatially controlled by other regulatory proteins, especially protein kinases^10^,^11^,^12^,^13^,^14^, and dysfunctions or dysregulations of autophagy receptors caused by gene mutations of autophagy receptors or related regulatory proteins are directly linked with many human diseases such as infectious diseases and neurodegenerative diseases^5^,^15^,^16^,^17^,^18^,^19^,^20^. However, the detailed molecular mechanism governing the specific interactions between autophagy receptors and their regulatory proteins, as well as the mechanistic explanations to the identified disease-associated mutations are still elusive.

OPTN is an ubiquitin-binding autophagy receptor, which is initially found to participate in the xenophagy and aggrephagy processes^10^,^21^, and more recently is also demonstrated to involve in the PARKIN-dependent mitophagy^11^,^22^,^23^. The predicted domain structure of OPTN includes two coiled-coil domains, a canonical LIR motif, an ubiquitin-binding UBAN domain that preferentially binds to linear and K63-linked ubiquitin chains^24^,^25^, and a C-terminal C_2_H_2_-type zinc finger (ZnF) with unknown function (Fig. 1a). The N-terminal coiled-coil region of OPTN is responsible for its interaction with TANK-binding kinase 1 (TBK1), which is a noncanonical IκB kinase family member involved in innate immunity and autophagy^10^,^11^,^26^,^27^. Interestingly, the specific interaction between OPTN and ATG8 proteins can be regulated by TBK1, which directly phosphorylates a serine residue immediately preceding the canonic LIR motif of OPTN to increase the binding affinity of OPTN with ATG8 family proteins, thereby promoting autophagic clearance of cellular invading *Salmonella*^10^. Furthermore, the UBAN-mediated ubiquitin recognition of OPTN is recently demonstrated to be tuned by TBK1 during the depolarization-dependent mitophagy^11^,^14^. In particular, the phosphorylation of Ser473 residue located within the UBAN of OPTN by TBK1 enhances the binding ability of OPTN to poly-ubiquitin chains, and promotes the efficiency of OPTN-involved mitophagy^11^,^14^. Therefore, the specific functions of OPTN in selective autophagy are well regulated by TBK1. However, how TBK1 associates with and phosphorylates OPTN is currently not known, and the detailed binding mechanism between OPTN and TBK1 remains to be elucidated.

In recent years, genetic variations of OPTN received particular attention due to the crucial pathophysiological roles of defective OPTN in many neurodegenerative diseases such as primary open-angle glaucoma (POAG) and amyotrophic lateral sclerosis (ALS), two progressive neurological disorders characterized by degenerations of retinal ganglion cells and motor neurons, respectively^18^,^28^,^29^,^30^. Among the OPTN mutations, the glaucoma-associated E50K mutation is demonstrated to associate with a more progressive and severe disease phenotype, and causes death of retinal ganglion cells in transgenic mice^31^,^32^. Importantly, more recent studies revealed that genetic defects of *TBK1,* in particular, a TBK1 E696K missense variant and a deletion mutation lacking part of the TBK1 C-terminal domain (hereafter referred to as CTD) that is necessary to interact with adaptor proteins, including OPTN, NAP1, TANK and SINTBAD^26^,^33^, are implicated in ALS pathogenesis^19^,^20^. Notably, although the OPTN E50K mutation was proved to increase the binding ability of OPTN towards TBK1 (ref. 26), and the TBK1 E696K variation was reported to abrogate the interaction between TBK1 and OPTN^19^, the underlying mechanism governing the neurodegenerative diseases caused by these mutations are poorly understood.

Here we biochemically and structurally characterize the interaction between OPTN and TBK1, and demonstrate that the direct association of OPTN N-terminal coiled-coil domain (NTD) with TBK1 CTD domain is responsible for the OPTN/TBK1 complex formation. The structures of OPTN NTD in complex with TBK1 CTD and the related NAP1/TBK1 complex solved in this study not only elucidate the detailed molecular mechanism underlying the OPTN and TBK1 interaction but also uncover a general interaction mode governing TBK1's binding to all of its currently identified binding adaptor proteins. Importantly, our structural data also provide mechanistic explanations to why several mutations found in OPTN and TBK1 can cause neurodegenerative diseases. Together, our findings are valuable for understanding the functions of OPTN and TBK1 in selective autophagy, as well as the pathogenesis of neurodegenerative diseases caused by mutations of OPTN and TBK1.

## Results

### The interaction regions between OPTN and TBK1

**Figure 1:**
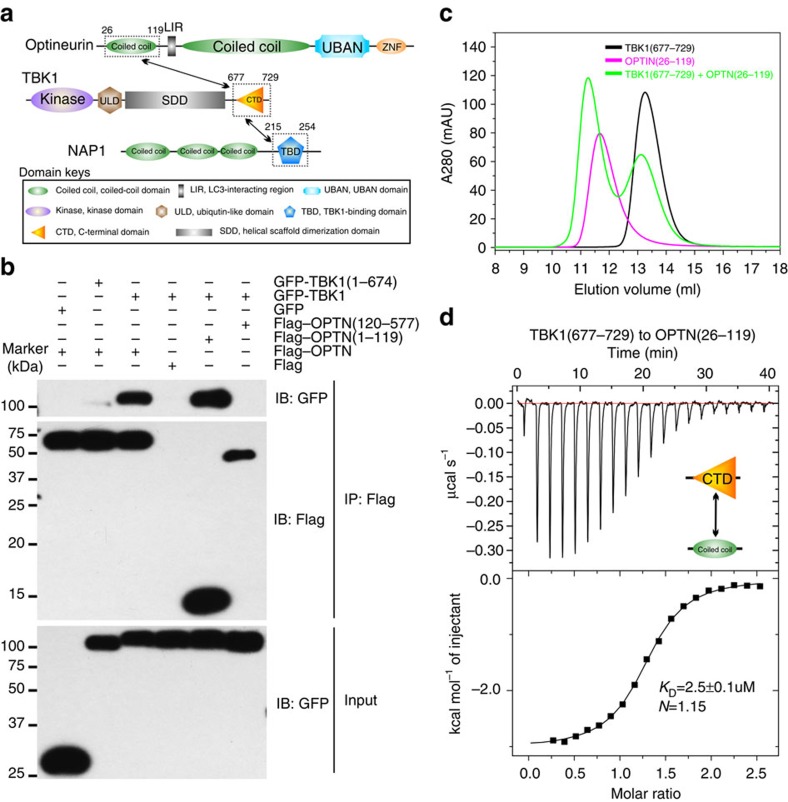
Biochemical characterization of the OPTN/TBK1 interaction. (**a**) A schematic diagram showing the domain organizations of OPTN, TBK1 and NAP1. In this drawing, the OPTN/TBK1 and NAP1/TBK1 interactions are further highlighted and indicated by two-way arrows. (**b**) Co-IP assay to map the detailed interaction between TBK1 and OPTN, showing that the CTD (residues 677–729) of TBK1 and the N-terminal region (residues 1–119) of OPTN are responsible for their interaction. (**c**) Analytical gel filtration chromatography analyses of the interaction between TBK1 CTD and OPTN(26–119). (**d**) ITC-based measurement of the binding affinity of TBK1 CTD with the OPTN(26–119) fragment. The *K*_D_ error is the fitted error obtained from the data analysis software, when using the one-site binding model to fit the ITC data. The ITC-measured *N* value, which is related to the binding stoichiometry, indicates that the binding stoichiometry of TBK1 CTD and OPTN(26–119) interaction is 1:1.

Previously, it was reported that the N-terminal region of OPTN could bind to the C-terminal part of TBK1 to mediate the OPTN/TBK1 complex formation^26^ (Fig. 1a). To further validate and define the specific interaction between OPTN and TBK1, we first carried out a co-immunoprecipitation (Co-IP) assay using different fragments of OPTN and TBK1 (Fig. 1b). Our results showed that TBK1 could interact with the full-length OPTN and the OPTN(1–119) fragment, whereas deletion of either the C-terminal region (residues 675–729) of TBK1 or the N-terminal region (residues 1–119) of OPTN completely abolished the OPTN/TBK1 complex formation (Fig. 1b). To further narrow down the TBK1-binding region in OPTN, we purified the recombinant proteins of two OPTN N-terminal fragments, OPTN(1–119) and OPTN(26–119), and used analytical gel filtration chromatography assay to analyse their interactions with TBK1 CTD (residues 677–729). The obtained result showed that both OPTN fragments could directly and specifically interact with TBK1 CTD (Supplementary Fig. 1a and Fig. 1c). In addition, isothermal titration calorimetry (ITC)-based quantitative analyses of the interactions between the two OPTN N-terminal fragments and TBK1 CTD revealed that the OPTN(1–119) and OPTN(26–119) fragments bind to TBK1 CTD with essentially the same *K*_D_ value, about 2.5 μM (Supplementary Fig. 1b and Fig. 1d). Importantly, detailed sequence alignment analyses of OPTN and TBK1 from different species revealed that the NTD (residues 36–119) of OPTN and the CTD (residues 679–729) of TBK1 are highly conserved, in line with their important roles to associate with each other (Supplementary Fig. 1c,d). Taken together, all the above biochemical data demonstrated that the highly conserved NTD of OPTN is sufficient for binding to TBK1 CTD.

### Overall structure of OPTN NTD in complex with TBK1 CTD

**Figure 2:**
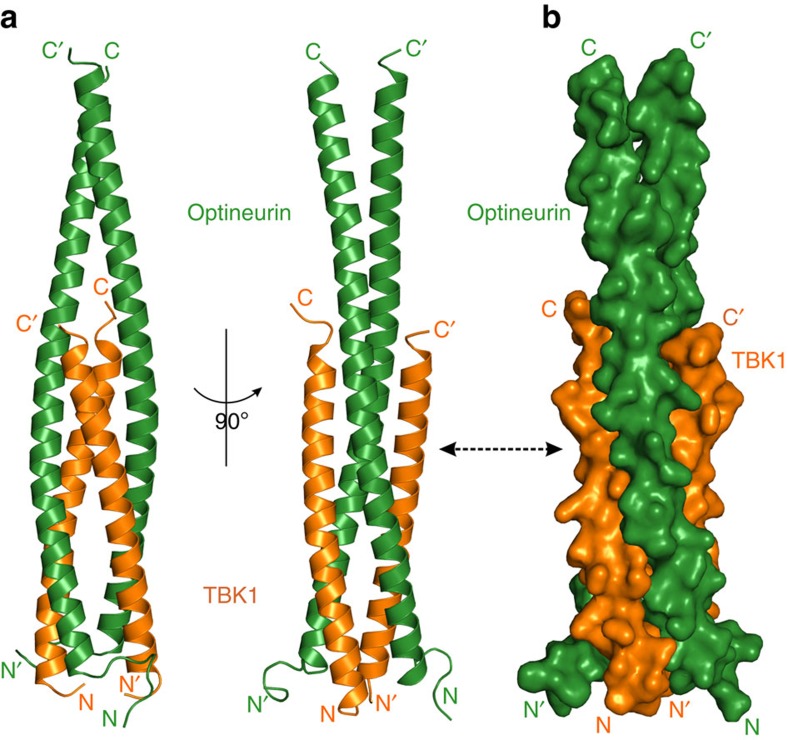
Overall structure of OPTN NTD in complex with TBK1 CTD. (**a**) Ribbon diagram showing the overall structure of OPTN(26–103)/TBK1 CTD complex. In this drawing, OPTN(26–103) is shown in forest green, TBK1 CTD in orange. (**b**) The surface representation showing the overall architecture of OPTN(26–103)/TBK1 CTD complex with the same colour scheme as in **a**.

To uncover the detailed molecular mechanism underpinning the specific interaction between OPTN and TBK1, we sought to determine the structure of OPTN NTD in complex with TBK1 CTD. Initially, we conducted a crystal screen using a complex formed by the OPTN(26–119) fragment and TBK1 CTD, and successfully obtained crystals that diffracted to 2.5 Å. Then, we determined the structure of this OPTN(26–119)/TBK1 CTD complex using the single-wavelength anomalous diffraction method with a Se-Met derivative data set. Unfortunately, in the crystal structure of OPTN(26–119)/TBK1 CTD complex, we found that the electron densities of the last 16 residues of OPTN coiled-coil region are very bad, and the conformations of these residues are unable to be well defined. Therefore, we made a short OPTN fragment, OPTN(26–103), by deleting the last 16 amino acids. Further ITC-based analysis confirmed that the OPTN(26–103) and OPTN(26–119) fragments have a similar binding affinity towards TBK1(677–729), and their binding modes with TBK1(677–729) are essentially the same (Fig. 1d and Supplementary Fig. 1e). Fortunately, using this short OPTN fragment in complex with TBK1 CTD, we obtained high-quality crystals that diffracted to 2.05 Å resolution. The OPTN(26–103)/TBK1 CTD complex structure was solved by the molecular replacement method using the OPTN(26–119)/TBK1 CTD structure as the search mode (Supplementary Table 1 and Supplementary Fig. 2a). In the final refined structural model, each asymmetric unit contains one OPTN(26–103)/TBK1 CTD complex molecule, which has a 2:2 stoichiometry and forms a symmetrical hetero-tetramer consisting of a OPTN dimer and two TBK1 CTD molecules (Fig. 2a). The OPTN(26–103)/TBK1 CTD complex adopts an overall elongated and atypical parallel four-helix bundle architecture (Fig. 2a). In the complex structure, the two OPTN molecules mainly form two crescent-shaped continuous α-helices with their C-terminal parts (residues 73–102) directly packing with each other to form a homo-dimeric interface and N-terminal parts head-to-head bridging across two TBK1 CTD molecules, each of which also adopts an α-helical configuration (Fig. 2a,b). Notably, the extreme C-terminal 11 amino acids (residues 719–729) of TBK1 CTD could not be traced in the complex structure, presumably due to the dynamic nature of this region.

A structural homology search using the Dali server^34^ revealed that the overall architecture of OPTN/TBK1 complex shared some similarities with the previously reported NEMO/IKKβ complex^35^ (Supplementary Fig. 2b–d). However, there are obvious differences between these two complexes. For instance, in contrast to the TBK1 CTD in the OPTN/TBK1 complex, the IKKβ molecule in the NEMO/IKKβ complex does not form continuous α-helix but contains a unique unwound C-terminal region, and directly packs with the middle parts of the NEMO helices; the dimerization of NEMO molecules in the NEMO/IKKβ complex is mediated by both N-terminal and C-terminal regions of NEMO, while the dimerization of OPTN in the OPTN/TBK1 complex only relies on its C-terminal helical part (Supplementary Fig. 2b–d). Therefore, the OPTN/TBK1 complex solved in this study represents a novel assembling mode for a parallel four-helix bundle formation.

### The molecular interface of the OPTN NTD/TBK1 CTD complex

**Figure 3:**
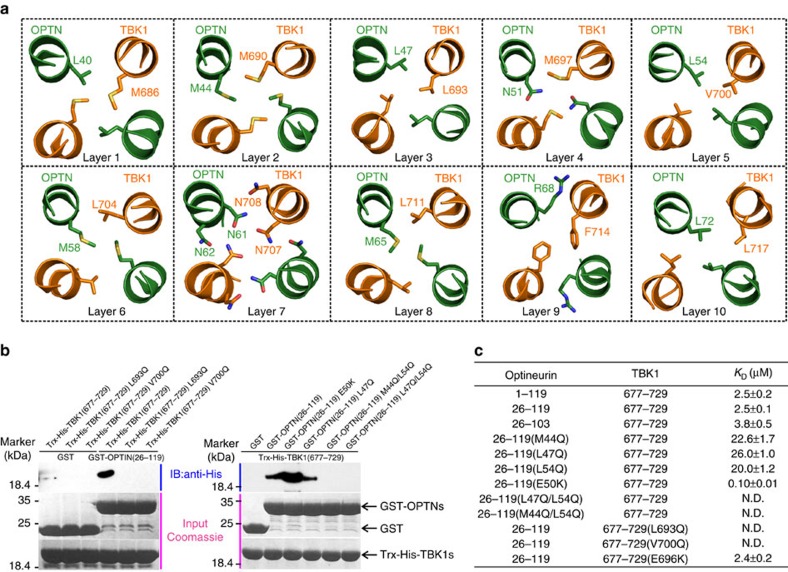
The molecular interface of the OPTN(26–103)/TBK1 CTD complex. (**a**) Ribbon-stick diagram showing the detailed interior interactions of the OPTN(26–103)/TBK1 CTD complex formed by 10 layers of interacting amino-acid side chains. (**b**) GST pull-down assays to verify the specific interaction between OPTN NTD and TBK1 CTD, as well as the key binding interface residues. The left panel showing the GST pull-down assay using purified GST-tagged OPTN(26–119) protein and various TBK1 CTD proteins, confirming the crucial interface residues L693 and V700 of TBK1 in binding to OPTN. The right panel showing the GST pull-down assay using purified TBK1 CTD protein and various GST-tagged OPTN(26–119) proteins, confirming the important interface residues M44, L47 and L54 of OPTN in binding to TBK1 CTD. (**c**) The measured binding affinities between various forms of OPTN and TBK1 proteins or their mutants by ITC-based binding assays. ‘ND' stands for that the *K*_D_ value is not detectable. The *K*_D_ errors are the fitted errors obtained from the data analysis software, when using the one-site binding model to fit the ITC data. The measured *N* value is related to the binding stoichiometry.

In the determined OPTN/TBK1 complex structure, there are two distinct interaction interfaces, the OPTN/TBK1-binding interface and the OPTN C-terminal dimerization interface (Fig. 2a,b). The binding interface between OPTN and TBK1 is within the N-terminal four-helix bundle core, whose interior is formed by 10 layers of interacting amino-acid side chains (Fig. 3a). Detailed structural analyses revealed that the assemblies of the 10 layers are mainly mediated by hydrophobic and polar interactions. In particular, the first, second, third, fifth, sixth, eighth, ninth and tenth layers are all formed by hydrophobic interactions, the hydrophilic fourth layer is maintained by a weak hydrogen bond between the side chain sulfur atom of TBK1 M697 residue and the side chain amide group of OPTN N51, and the unique seventh layer is formed by a hydrogen bond interaction network between the polar side chains of TBK1 N707, N708 residues and the side chains of N61, N62 from OPTN (Fig. 3a). In addition, the OPTN/TBK1 complex is further stabilized by extensive surface interactions between OPTN and TBK1 (Supplementary Fig. 3a,b). Importantly, all the key residues involved in the binding interface either from OPTN or TBK1 are highly conserved during evolution (Supplementary Fig. 1c,d). In the complex structure, the C-terminal coiled-coil region (residues 73–102) of OPTN consists of four heptad repeats, and is responsible for the OPTN dimer formation. The dimerization of OPTN was mediated by both hydrophobic and polar interactions between residues located in the *a*, *c*, *d* and *g* positions of the two OPTN helices (Fig. 2a and Supplementary Fig. 3c).

Using the glutathione-*S*-transferase (GST)-fusion protein affinity pull-down assay and the quantitative ITC-based analysis, we verified the specific interaction between the OPTN NTD and the TBK1 CTD. Consistent with the structural data, single-point mutations of the key residues located at the binding interface of OPTN, such as M44Q, L47Q and L54Q, all largely attenuated the OPTN/TBK1 complex formation, and reduced the binding affinity *K*_D_ values to about 22.6, 26.0, and 20.0 μM, respectively (Fig. 3b,c and Supplementary Fig. 4a–c). Furthermore, double mutations of the key interface residues of OPTN such as M44Q/M54Q, L47Q/L54Q of OPTN or, conversely, single-point mutations of the key interface residues of TBK1, such as L693Q and V700Q of TBK1, all essentially abolished the specific interaction between OPTN and TBK1 (Fig. 3b,c and Supplementary Fig. 4d–g). Finally, using cellular functional assay we also demonstrated that the specific interaction between OPTN NTD and TBK1 CTD was necessary not only for the co-localization of OPTN and kinase-dead TBK1 in heterologous cells (Supplementary Fig. 5), but also for the OPTN-mediated autophagic degradation of GFP-polyQ aggregates in transfected HeLa cells (Supplementary Fig. 6).

### TBK1 interacts with adaptors using a similar binding mode

In addition to OPTN, the C-terminal domain of TBK1 was also implicated in the binding to several adaptor proteins, including NAP1, TANK and SINTBAD^33^. Particularly, NAP1 likely mediates the recruitment of TBK1 to the autophagy receptor NDP52, as it can simultaneously interact with NDP52 and TBK1 (ref. 36). To further investigate whether TBK1 binds to different adaptor proteins using a similar binding mode, and whether different adaptor proteins are competitive or cooperative in binding to TBK1, we sought to elucidate the detailed binding mechanism between NAP1 and TBK1. Careful sequence alignment analysis showed that the TBK1-binding domain (TBD) of NAP1 (residues 215–254) is highly conserved during the evolution and also predicted to form a helical structure (Supplementary Fig. 7a). Analytical gel filtration chromatography and ITC-based assays revealed that NAP1 TBD can directly interact with the TBK1 CTD with a binding affinity *K*_D_ value of ∼0.72 μM (Supplementary Fig. 7b,c), which is roughly fourfold stronger than that of the OPTN/TBK1 complex (Fig. 3c). To further uncover the detailed interaction mode between TBK1 and NAP1, we also determined the high-resolution crystal structure of the NAP1 TBD/TBK1 CTD complex (Supplementary Table 2 and Supplementary Fig. 7d).

**Figure 4:**
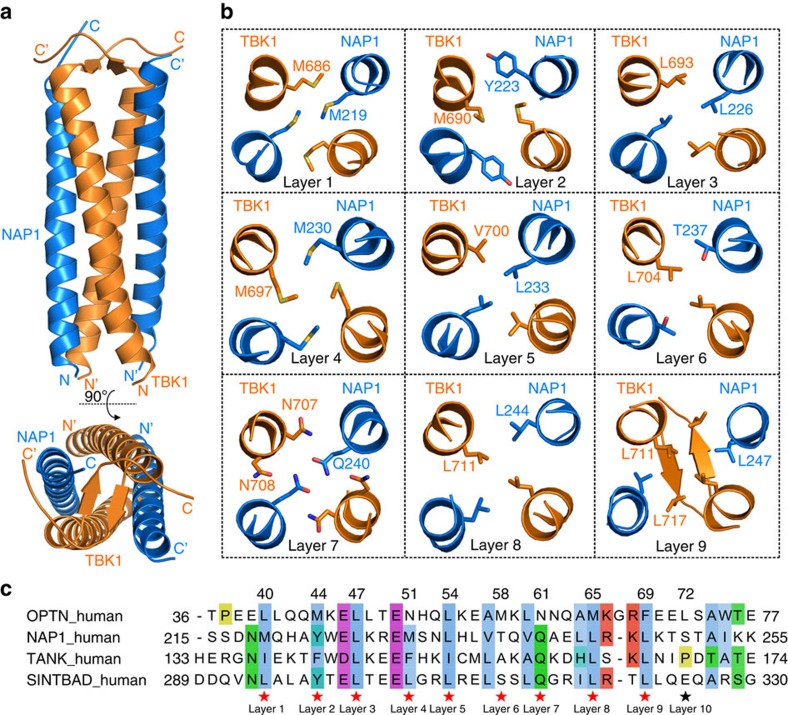
A similar interaction mode shared by TBK1-binding adaptor proteins in binding to TBK1 CTD. (**a**) Ribbon diagram showing the overall structure of NAP1 TBD/TBK1 CTD complex. In this drawing, the NAP1 TBD is shown in blue and TBK1 CTD in orange. (**b**) Ribbon-stick diagram showing the detailed interior interactions of the NAP1 TBD/TBK1 CTD complex formed by nine layers of interacting amino-acid side chains. (**c**) The detailed sequence alignment of TBK1-binding regions in current known four TBK1-binding adaptors, OPTN, NAP1, TANK and SINTBAD. In this alignment, the conserved residues are highlighted by colours using software Jalview2.8.1 (http://www.jalview.org/). The key residues of OPTN and NAP1 that mediated the interactions with TBK1 to assemble the internal layer structures are further highlighted with red stars except the unique hydrophobic residue of OPTN for the tenth layer formation, which is only found in OPTN and highlighted with a black star.

As expected, the NAP1 TBD/TBK1 CTD complex structure consists of two NAP1 TBD and two TBK1 CTD molecules, which pack together to form a symmetrical hetero-tetramer (Fig. 4a). The hetero-tetrameric NAP1 TBD/TBK1 CTD complex mainly adopts a parallel and compact four-helix bundle architecture, which is capped by a short anti-parallel β-sheet formed by the C-terminal ends of two TBK1 CTD molecules (Fig. 4a). The overall structure of NAP1 TBD/TBK1 CTD complex is very similar to the four-helix bundle core region of OPTN/TBK1 complex (Supplementary Fig. 8a). However, in contrast to its continuous helical structure in the OPTN/TBK1 complex, the C-terminal region (residues 715–717) of TBK1 CTD in the NAP1/TBK1 complex adopts a short β-strand, which directly augments with the β-strand of the counterpart CTD molecule in an anti-parallel manner (Supplementary Figs 1d and 8a). In addition, unlike the dimeric OPTN coiled-coil domains in the OPTN/TBK1 complex, there is no dimerization site between two NAP1 helices in the NAP1/TBK1 complex (Fig. 4a and Supplementary Fig. 8a). Further detailed structure analysis showed that the interior of the NAP1/TBK1 complex is stabilized by nine layers of interacting amino-acid side chains that are mainly mediated by hydrophobic contacts and polar interactions (Fig. 4b). In particular, the hydrophobic side chain of NAP1 L247 buries in a hydrophobic pocket formed by the side chains of L711 and L717 residues from TBK1 to assemble the unique hydrophobic ninth layer, which is further stabilized by a pair of backbone hydrogen bonds formed between two S716 residues located in the anti-parallel β-sheet of TBK1 (Fig. 4b). In addition, extensive hydrophobic and polar interactions located at the external surface of the four-helix bundle further contribute to the NAP1/TBK1 complex formation (Supplementary Fig. 8b,c). Using ITC-based analysis, we also verified the specific interaction between NAP1 TBD and TBK1 CTD. In line with the structural data, individual point mutations of key interface residues either from NAP1 or TBK1, such as the L226Q, L233Q, L244Q mutations of NAP1 or the L693Q, V700Q mutations of TBK1, all largely decreased or completely abrogated the NAP1 TBD/TBK1 CTD complex formation (Supplementary Figs 7e and 9a–e).

Detailed structural comparison of the NAP1/TBK1 and OPTN/TBK1 complexes revealed that TBK1 used the same residues (M686, M690, L693, M697, V700, L704, N707, N708, L711 and L717) to assemble the internal layer structures of these two complexes, except for the F714 residue that is only important for the ninth layer formation in the OPTN/TBK1 complex (Figs 3a and 4b). Consistent with this structural observation, further biochemical analyses demonstrated that the OPTN NTD and NAP1 TBD domain are competitive in binding to TBK1 CTD (Supplementary Fig. 10). Moreover, a detailed sequence alignment analysis of the identified TBK1-binding motifs in currently known TBK1-binding adaptors including OPTN, NAP1, TANK and SINTBAD showed that key residues that are important for binding with TBK1 to assemble the internal first, second, third, fifth, seventh, eighth and ninth layers are highly similar among the TBK1-binding adaptors, although the residues for the fourth and sixth layers are relatively variable and the hydrophobic residue for the tenth layer can be only found in OPTN (Fig. 4c). Given these structural and sequence analyses, we inferred that these TBK1-binding adaptors likely share a similar binding model and competitively to interact with TBK1 to form distinct TBK1/adaptor complexes, which are presumably responsible for different intracellular signalling pathways.

### Mechanistic insights into the OPTN E50K mutation

**Figure 5:**
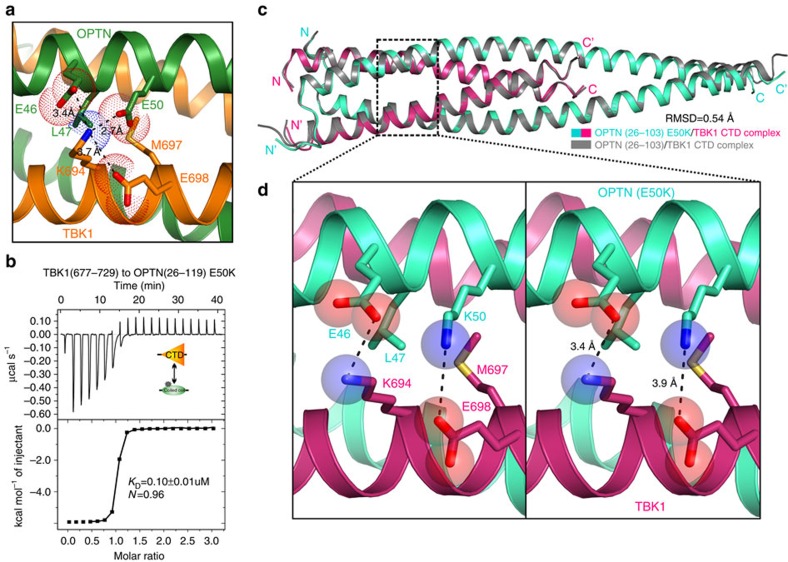
Mechanistic insights into the POAG-associated OPTN E50K mutation on the OPTN and TBK1 interaction. (**a**) The combined ribbon and the stick-dot representations showing the detailed roles of OPTN E50 residue in the OPTN NTD and TBK1 CTD interaction. In this drawing, the side chains of key residues are shown in the stick mode, and the salt bridges, hydrogen bonds and charge–charge interactions are indicated by black dash lines. (**b**) The ITC-based measurement of the binding affinity of OPTN(26–119) E50K in binding to TBK1 CTD. The *K*_D_ error is the fitted error obtained from the data analysis software, when using the one-site binding model to fit the ITC data. The measured *N* value is related to the binding stoichiometry. (**c**) Superposition ribbon diagram showing the comparison of the overall structures of the OPTN(26–103)/TBK1 CTD complex (grey) and the OPTN(26–103) E50K/TBK1 CTD mutant complex (colourized). (**d**) Enlarged stereo view showing the detailed structural role of OPTN K50 residue in the OPTN(26–103) E50K/TBK1 CTD mutant complex. In this drawing, the side chains of key residues are shown in the combined ribbon and stick-sphere mode, and the related hydrogen bonds, salt bridges and charge–charge interactions are indicated by black dash lines.

Mutation of OPTN was known to associate with ALS and POAG^18^,^28^,^29^,^37^. So far, a total of five missense mutations within the N-terminal region of OPTN (residues 1–119) have been documented in human patients, of which the R96L mutation is associated with ALS, and the H26D, E50K, M98K and E103D alterations are linked with POAG^30^,^37^ (Supplementary Fig. 1c). On the basis of the determined OPTN(26–103)/TBK1 CTD complex structure, the H26, R96, M98 and E103 residues do not locate in the TBK1-binding region of OPTN. Therefore, the H26D, R96L, M98K and E103D mutations are unlikely to affect the interaction between OPTN and TBK1 but instead are likely to interfere the oligomeric state of OPTN or the interaction between OPTN and other unknown binding partners. In the OPTN(26–103)/TBK1 CTD complex structure, the OPTN E50 residue is exposed to the solvent and plays an adjuvant role for the OPTN/TBK1 interaction by forming a charge–charge interaction with the positively charged TBK1 K694 residue, which is further neutralized by two nearby negatively charged OPTN E46 and TBK1 E698 residues (Fig. 5a). Obviously, the E50K mutation would disrupt the salt bridge interaction seen in the wild-type OPTN/TBK1 complex, but, surprisingly, the OPTN E50K mutant shows a roughly 43-fold increase of binding affinity towards TBK1 compared with the wild-type protein (Figs 1d and 5b), consistent with our Co-IP result (Supplementary Fig. 11d) and several earlier observations that the E50K mutation would increase the interaction between OPTN and TBK1 in cells^26^,^38^. To further elucidate the detailed mechanism, we also solved the structure of OPTN(26–103) E50K mutant in complex with TBK1 CTD (Supplementary Table 2).

**Figure 6:**
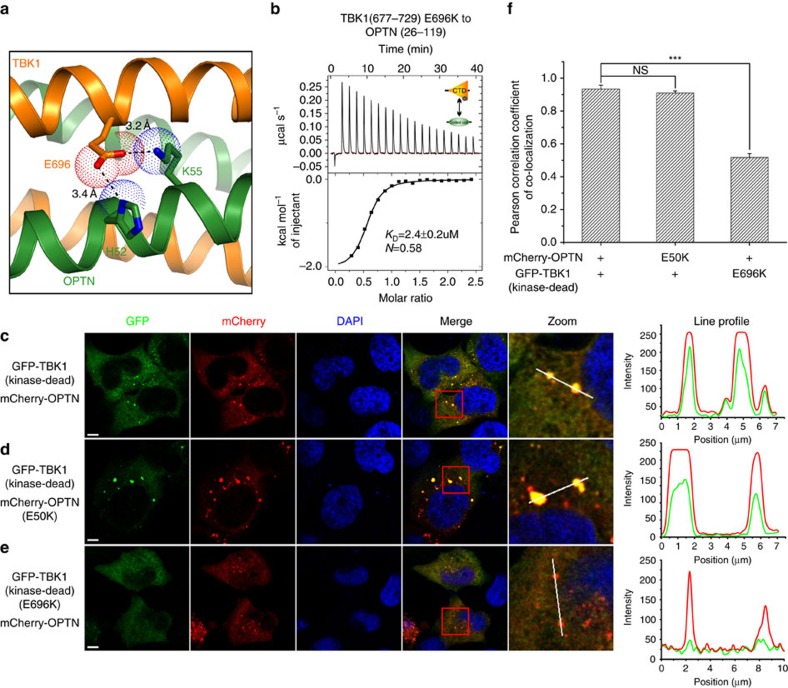
The effects of ALS-associated TBK1 E696K mutation on the formation of OPTN/TBK1 complex. (**a**) The combined ribbon and the stick-dot representations showing the detailed roles of ALS-associated TBK1 E696 residue in the TBK1 CTD and OPTN NTD interaction. In this drawing, the side chains of key residues are shown in the stick mode, and the related hydrogen bonds and salt bridges are indicated by black dash lines. (**b**) The ITC-based measurement of the binding affinity of the OPTN(26–119)/TBK1 CTD(E696K) complex. The *K*_D_ error is the fitted error obtained from the data analysis software, when using the one-site binding model to fit the ITC data. The obtained *N* value indicates that the binding stoichiometry of TBK1 CTD(E696K) and OPTN(26–119) interaction is 1:2. (**c**–**e**) The cellular co-localization analyses of OPTN, kinase-dead TBK1, as well as the neurodegenerative diseases-associated OPTN E50K and TBK1 E696K mutants in transfected HeLa cells. Since overexpression of wild-type TBK1 in transfected HeLa cells markedly induces cell death with unknown reasons, thereby, all the related TBK1 mutants used in the co-localization experiments were derived from the kinase-dead TBK1 S172A mutant. (**c**) When co-transfected, the kinase-dead TBK1 co-localizes very well with the OPTN clusters in cells. (**d**) The POAG-associated OPTN E50K mutation induces the formations of large OPTN puncta, which perfectly co-localize with the kinase-dead TBK1 puncta in co-transfected cells. (**e**) The ALS-causative TBK1 E696K mutation largely decreases the co-localization of kinase-dead TBK1 with the OPTN puncta in co-transfected cells. (**f**) Statistical results related to the co-localizations of OPTN and kinase-dead TBK1, as well as their mutants in the co-transfected HeLa cells shown as Pearson correlation. The Pearson correlation coefficient analysis was performed using the LAS X software based on a randomly selected region that roughly contains one co-transfected HeLa cell. The data represent mean±s.d. of >50 analysed cells (selected regions) from two independent experiments, and scale bar, 5 μm. The unpaired Student's *t*-test analysis was used to define a statistically significant difference, and the asterisks indicate the significant differences between the indicated bars (***P<0.001) and NS stands for not significant.

To our surprise, the overall structure of the mutant complex is almost the same as that of the OPTN(26–103)/TBK1(677–729) complex (Fig. 5c). However, detailed structural analysis revealed that the positively charged OPTN K50 residue in the mutant complex forms a charge–charge interaction with the negatively charged TBK1 E698 residue, and meanwhile its long aliphatic side chain packs extensively with the hydrophobic pocket formed by the side chain of OPTN L47 and TBK1 M697 residues (Fig. 5d). Given that the OPTN/TBK1 complex is a symmetrical tetramer, these additional synergic interactions induced by the E50K mutation likely contribute to the increase of binding affinity observed between OPTN E50K mutant and TBK1. Interestingly, multi-angle light scattering (MALS) analysis revealed that the E50K mutation could change the oligomeric state of OPTN N-terminal region (residues 1–119) and induce its tetramerization (Supplementary Fig. 11a). In line with aforementioned structural and biochemical analyses, the cellular localization assay showed that the OPTN E50K mutant formed much larger puncta when compared with the wild-type protein, and completely co-localized with the kinase-dead TBK1 puncta in co-transfected HeLa cells (Fig. 6c,d,f). Collectively, these data demonstrated that the POAG-associated OPTN E50K mutation not only enhances the interaction between OPTN and TBK1 but also affects the oligomeric state of OPTN.

### The effects induced by TBK1 E696K mutation

Recently, *TBK1* was also demonstrated to be an ALS-causative gene^19^,^20^. Particularly, an E696K missense variant, which was reported to abrogate the interaction between TBK1 and OPTN in cells, was showed to be involved in ALS pathogenesis^19^. According to the structure of OPTN(26–103)/TBK1 CTD complex, the TBK1 E696 residue locates at the external surface of the four-helix bundle core, and forms two side chain hydrogen bonds with the H52 and K55 residues of OPTN (Fig. 6a). Apparently, the substitution of E696 with a positively charged Lys residue would disrupt those interactions, thereby disturbing the OPTN/TBK1 complex formation. Interestingly, further GST-fusion protein pull-down assay and ITC measurement revealed that the E696K mutation of TBK1 did not abolish the interaction between OPTN(26–119) and TBK1 CTD *in vitro* (Supplementary Fig. 11b and Fig. 6b). However, the ITC data showed that the measured *N* values, which are related to the binding stoichiometry, of OPTN/TBK1 interaction and OPTN/TBK1 E696K mutant interaction are 1.15 and 0.58, respectively (Figs 1d and 6b), indicating that the TBK1 E696K mutation would alter the binding mode between TBK1 and OPTN. Indeed, further MALS-based analysis demonstrated that the oligomeric condition of TBK1 CTD was largely affected by the E696K mutation (Supplementary Fig. 11c). Intriguingly, in contrast to the *in vitro* biochemical results, cellular co-localization assay showed that the TBK1 E696K mutation largely diminished the co-localization between OPTN and kinase-dead TBK1 in the co-transfected cells (Fig. 6c,e,f), and further Co-IP experiments revealed that the TBK1 E696K mutation almost totally abolished the specific interaction between OPTN and TBK1 in transfected cells (Supplementary Fig. 11d), consistent with two recent reports^14^,^19^. However, the exact reasons for the observed discrepancy of TBK1 E696K mutation on the interaction of OPTN and TBK1 *in vitro* and *in vivo* are yet to be elucidated. Interestingly, additional ITC and Co-IP assay showed that the TBK1 E696K mutation did not disrupt the interaction between TBK1 and NAP1 *in vitro* or in cells (Supplementary Figs 9f and 11e). Therefore, the ALS-associated TBK1 E696K mutation only specifically affected the OPTN/TBK1 interaction, and did not have a general effect on other TBK1–adaptor interactions.

## Discussion

In the present study, we biochemically and structurally characterized the interactions between TBK1 and two related adaptor proteins, OPTN and NAP1, and revealed that TBK1 uses a similar interaction mode and almost the same key residues within its CTD domain to associate with OPTN and NAP1. Accordingly, OPTN and NAP1 are mutually exclusive in binding to TBK1, and likely to form distinct TBK1/adaptor complexes for different intracellular signalling pathways. In addition to TBK1, NAP1 was also reported to directly interact with the autophagy receptor NDP52 and TAX1BP1 through its N-terminal coiled-coil region^36^, thereby serving as an adaptor to couple NDP52 or TAX1BP1 with TBK1. Therefore, we speculated that TBK1 likely utilized two different approaches, directly and indirectly, to associate with autophagy receptor OPTN and NDP52 (or TAX1BP1), respectively. Interestingly, previous studies indicated that TBK1, together with OPTN, NDP52 and SQSTM1, was recruited to the ubiqutinated pathogen in xenophagy, as well as the damaged mitochondria in the depolarization-dependent mitophagy, and cooperated with OPTN, NDP52 and SQSTM1 in those selective autophagy processes^10^,^11^,^14^,^36^. Particularly, TBK1 could directly phosphorylate OPTN to regulate its interactions with ATG8 family proteins as well as ubiquitin proteins^10^,^11^,^14^, and SQSTM1 to facilitate its associations with ubiquitinated cargoes^39^. However, which TBK1/adaptor complex is associated with SQSTM1 and responsible for its phosphorylation remains to be elucidated. Strikingly, although TBK1 could also phosphorylate NDP52 (refs 11, 14), the precise downstream effects of TBK1-mediated phosphorylation of NDP52 in selective autophagy are still unknown. Furthermore, the activation of TBK1 in response to mitochondrial depolarization in mitophagy was proved to involve both OPTN and NDP52 as well as the poly-UB binding ability of OPTN^11^,^23^. So far, the exact activation mechanism of TBK1 mediated by OPTN and NDP52 in selective autophagy is still poorly understood. Therefore, additional studies are required to elucidate the detailed molecular mechanism governing the mutual regulations between TBK1 and autophagy receptors.

Previous studies implied that mutations of OPTN, such as the OPTN E50K missense mutation, are directly linked to POAG disease. In this study, we demonstrated that the OPTN E50K mutation not only enhanced the binding between OPTN and TBK1 but also altered the oligomeric state of OPTN *in vitro*, as well as in cells. Therefore, at least two aspects of functional property of OPTN are affected by the disease-causing E50K mutation, and both loss-of-function and gain-of-function mechanisms are likely involved in the pathogenesis of neurodegenerative diseases caused by this OPTN mutation. Apparently, more studies, especially quantitative proteomic analyses, are required to dissect the *in vivo* targets of OPTN E50K mutation in motor neurons, thereby to clarify how E50K mutation found in OPTN is associated with human glaucoma disease.

In this study, we also elucidated that the ALS-associated TBK1 E696K mutation altered the binding mode between OPTN NTD and TBK1 CTD *in vitro*, and essentially abolished the interaction of TBK1 with OPTN *in vivo*. Interestingly, the TBK1 E696K mutation had little effect on the TBK1/NAP1 interaction. Therefore, the TBK1 E696K mutation likely only specifically inhibited the TBK1 and OPTN interaction, in line with a recent report^14^. Interestingly, many currently known ALS-linked OPTN mutations, including two missense mutations Q454E and E478G, as well as the truncation mutation Q398X, all located at the C-terminal part of OPTN rather than the N-terminal TBK1-binding region^37^. On the basis of our current structural data, those ALS-linked mutations located at the C-terminal part of OPTN are unlikely to affect the interaction between OPTN and TBK1, but more likely to disturb or disrupt the function of OPTN UBAN domain, which is critical for the OPTN-dependent selective autophagy processes^22^,^40^. Indeed, the ALS-linked OPTN E478G mutation was demonstrated to abolish the binding ability of OPTN UBAN to ubiquitin chains^10^,^18^, and disrupt the autophagic function of OPTN in mitophagy^22^. The function of OPTN in selective autophagy was proved to be regulated by TBK1 kinase, and particularly TBK1 can directly phosphorylate OPTN to promote the efficiency of OPTN-involved selective autophagy^10^,^11^,^14^. Importantly, during those processes, the OPTN-mediated recruitment of TBK1 to the ubiquitin-decorated cargoes such as damaged mitochondria, and the subsequent activation of TBK1 kinase activity, as well as the final phosphorylation of OPTN by TBK1 all rely on the specific interaction of OPTN with TBK1 and/or the proper function of OPTN UBAN domain^10^,^11^,^14^,^22^,^23^,^41^. Therefore, we speculated that dysfunctions of OPTN UBAN domain caused by those ALS-linked OPTN mutations such as E478G and defective interaction of TBK1 with OPTN induced by the ALS-linked TBK1 E696K mutation should lead to a common consequence, affecting the OPTN-dependent aggrephagy and mitophagy, which are two selective autophagy processes highly related to neurodegenerative diseases^16^,^42^. Thus, our study mechanistically linked, *OPTN* and *TBK1*, two genes mutated in ALS disease in a common selective autophagy pathway.

Finally, taking all the data together, we proposed a model depicting the recruitment of OPTN and TBK1 to ubiquitin-decorated dysfunctional mitochondria, as well as the regulation of OPTN by TBK1 in mitophagy (Supplementary Fig. 12). In this model, OPTN and TBK1 both formed dimers, and associated with each other to form stable hetero-tetrameric complexes mediated by the unique interaction between the OPTN NTD and the TBK1 CTD. Then the OPTN/TBK1 hetero-tetrameric complexes were recruited to the ubiqutinated mitochondria through the OPTN UBAN domains, which could specifically recognize the poly-ubiquitin chains decorated on the damaged mitochondria. Finally, after activation of TBK1, the activated TBK1 molecules directly phosphorylated OPTN either through an intermolecular or an intramolecular manner to enhance the binding abilities of OPTN to ATG8 family proteins and ubiquitin proteins, thereby amplifying the mitophagy process to promote autophagic clearance of dysfunctional mitochondria. We speculated that the properly spatial and temporal control of the TBK1 kinase activity, as well as the phosphorylation levels of its substrates, and an optimum level of OPTN-mediated selective autophagy are crucial for maintaining cellular homeostasis, especially in neurons, and impaired autophagy is likely to contribute to the pathogenesis of neurodegenerative diseases caused by defective OPTN and TBK1.

## Methods

### Materials

HEK293T, HeLa cell lines and GFP-htt140Q plasmid were kindly provided by Prof. Junying Yuan from Interdisciplinary Research Center on Biology and Chemistry, CAS, Shanghai, China. The full-length human TBK1 plasmid was a gift from Dr Jin Zhong from Institut Pasteur of Shanghai, CAS, Shanghai, China. The full-length human OPTN and NAP1 plasmids were obtained from Prof Jiahuai Han from School of Life Sciences, Xiamen University, Xiamen, China.

### Protein expression and purification

The different DNA fragments encoding human OPTN (residues 1–119, 26–119 and 26–103), TBK1 CTD (residues 677–729) and NAP1 (residues 215–254) were PCR amplified from the full-length human OPTN, TBK1 and NAP1 cDNA, respectively. All these fragments were either cloned into the pET-32M vector (a modified version of pET32a vector containing a N-terminal Trx-tag and His_6_-tag) or the pET-GST vector (a modified version of pET32a vector containing a N-terminal GST-tag) for recombinant protein expressions. For fluorescence imaging experiment, the DNA fragments encoding TBK1 (residues 1–729 and 1–674), OPTN (residues 1–119, 120–577 and 1–577) were cloned into pEGFP-C1 and pmCherry-C1 vectors, respectively, and these OPTN fragments were also cloned into the pCMV-flag vector for the Co-IP experiments. All the point mutations of OPTN, TBK1 and NAP1 used in this study were created using the standard PCR-based mutagenesis method, further checked by Taq Master mix (Vazyme Biotech Co., Ltd.) and confirmed by DNA sequencing.

Recombinant proteins were expressed in BL21 (DE3) *Escherichia coli* cells induced by 200 μM isopropyl-β-D-thiogalactoside (IPTG) at 16 °C. The bacterial cell pellets were resuspended in the binding buffer (50 mM Tris, 500 mM NaCl and 5 mM imidazole at pH 7.9), and then lysed by the ultrahigh pressure homogenizer FB-110XNANO homogenizer machine (Shanghai Litu Machinery Equipment Engineering Co., Ltd.). Then the lysis was spun down by centrifuge at 35,000*g* for 30 min to remove the pellets. His_6_-tagged proteins were purified by Ni^2+^-NTA agarose (GE Healthcare) affinity chromatography, while GST-tagged proteins were purified by glutathione sepharose 4B (GE Healthcare) affinity chromatography. Each recombinant protein was further purified by size-exclusion chromatography. The N-terminal tag of each recombinant protein was cleaved by 3C protease and further removed by size-exclusion chromatography. To prepare the SeMet-labelled protein, the cells were cultured in 1 l of M9 minimal medium at 37 °C until the OD_600_ reached at 0.5, and then supplied with 50 mg of SeMet (J&K Scientific Ltd, China) as the sole Met source, by additional adding 0.1 g of Lys, Thr and Phe, and 0.05 g of Leu, Ile and Val, to inhibit endogenous synthesis of Met. The protein expression was induced by the addition of IPTG to a final concentration of 200 μM, followed by further incubation at 16 °C overnight.

### Analytical gel filtration chromatography

Analytical gel filtration chromatography was carried out on an AKTA FPLC system (GE Healthcare). Protein samples were loaded on to a Superose 12 10/300 GL column (GE Healthcare) equilibrated with a buffer containing 20 mM Tris-HCl (pH 7.5), 100 mM NaCl and 1 mM dithiothreitol (DTT).

### Protein crystallization and structural elucidation

Crystals of OPTN(26–103)/TBK1 CTD complex and OPTN(26–103) E50K/TBK1 CTD complex were obtained by mixing the freshly purified complex protein (10 or 20 mg ml^−1^ in 20 mM Tris-HCl, 100 mM NaCl, 1 mM DTT and 1 mM EDTA at pH 7.5) with equal volumes of reservoir solution containing 0.1 M MES monohydrate (pH 6.0), 14% w/v PEG 4000 using the sitting-drop vapour-diffusion method at 16 °C. Before diffraction experiments, glycerol (10%) was added as the cryo-protectant. A 2.05 Å resolution X-ray data set for OPTN(26–103)/TBK1 CTD and a 2.5 Å resolution data set for OPTN(26–103) E50K/TBK1 CTD were collected at the beamline BL17U1 of the Shanghai Synchrotron Radiation Facility. The diffraction data were processed and scaled using HKL2000 (ref. 43).

To solve the phase problem, we obtained the Se-Met OPTN(26–119)/TBK1 CTD complex crystals in the condition of 0.2 M L-proline, 0.1 M HEPES (pH 7.0), 24% w/v PEG 1,500 and collected a 2.5 Å resolution X-ray data set. With the SeMet-derivative data set, the single-wavelength anomalous diffraction phase was determined and a partial structural model was traced using AutoSol^44^. The structure model was further built manually using COOT^45^ based on the experimental phase and then refined using PHENIX^44^. The phase problem of OPTN(26–103)/TBK1 CTD and OPTN(26–103) E50K/TBK1 CTD complexes were solved by molecular replacement method using the structure of the SeMet-OPTN(26–119)/TBK1 CTD complex with PHASER^46^. The initial model was rebuilt manually and then refined using REFMAC^47^. Further manual model building and adjustment were completed using COOT^45^. The qualities of the final model were validated by MolProbity^48^. In the final stage, an additional TLS refinement was performed in PHENIX^44^.

For the NAP1 TBD/TBK1 CTD complex structure, we first construct a fusion protein expression plasmid by directly fusing the NAP1(215–254) with the TBK1 CTD through a TEV cutting site. And then, the fusion protein was purified by the aforementioned standard method. 3C and TEV enzymes were added to cut the Trx-His tag and the linker, respectively. The complex protein was further purified by size-exclusion chromatography. The high-quality crystals of NAP1 TBD/TBK1 CTD complex were obtained by mixing the complex protein (20 mg ml^−1^ in 20 mM Tris-HCl, pH 7.5, 100 mM NaCl, 1 mM DTT and 1 mM EDTA) with equal volumes of reservoir solution containing 1% w/v tryptone, 0.05 M HEPES sodium (pH 7.0) and 12% w/v PEG 3350 using the sitting-drop, vapour-diffusion method for 2 weeks at 16 °C. Before diffraction experiments, glycerol (10%) was added as the cryo-protectant. A 1.5 Å resolution X-ray data set were collected at the Shanghai Synchrotron Radiation Facility. The data process and the structure refinement were the same as the OPTN/TBK1 complex. The phase problem was solved by molecular replacement method using the structure of OPTN(26–103)/TBK1 CTD complex with PHASER^46^. The final refinement statistics of solved structures in this study were listed in Supplementary Tables 1 and 2. Structural diagrams were prepared using the program PyMOL (http://www.pymol.org/).

### GST pull-down assay

Direct interactions between different OPTN and TBK1 fragments were analysed in the buffer containing 50 mM Tris (pH 7.5), 100 mM NaCl, 1 mM DTT and 1 mM EDTA. A unit of 50 μg of GST-tagged OPTN proteins and Trx-His-tagged TBK1 proteins were mixed at a molar ratio of 1:4 in 1 ml of the assay buffer. The different GST-OPTN /TBK1 complexes were pelleted by adding 30 μl of fresh glutathione sepharose 4B beads (GE Healthcare). The pellets were washed six times with 1 ml of the assay buffer and subsequently boiled with 30 μl of 2 × SDS–PAGE sample buffer. The proteins in the gel were visualized by Coomassive-blue staining or immuno-detection using anti-His antibody (Cell Signaling Technology, catalogue no. 2366, 1:3,000 dilutions). The results are summarized in Supplementary Fig. 13.

### The TBK1-binding competition assay

The competition assay was carried out using analytical gel filtration chromatography on an AKTA FPLC system (GE Healthcare). The purified OPTN(26–119)/TBK1(677–729) complex was incubated with increasing molar ratio of NAP1(215–254) protein, and then, the mixture samples were loaded on to a Superdex 200 increase 10/300 GL column (GE Healthcare) equilibrated with a buffer containing 20 mM Tris-HCl (pH 7.5), 100 mM NaCl and 1 mM DTT. The protein components in the corresponding fractions were analysed by SDS–PAGE and visualized by Coomassie-blue staining.

### ITC assay

ITC measurements were carried out on an ITC200 (GE Healthcare) or Microcal PEAQ-ITC (Malvern) calorimeter at 25 °C. All protein samples were in the same buffer containing 50 mM Tris (pH 7.5), 100 mM NaCl, 1 mM EDTA and 1 mM DTT. The concentrated 0.1 mM OPTN or NAP1 fragments and 1 mM of TBK1 fragment proteins were loaded into the cell and the syringe, respectively. The titration processes were performed by injecting 40 μl aliquots of the TBK1 proteins into OPTN or NAP1 fragments at time intervals of 2 min to ensure that the titration peak returned to the baseline. The titration data were analysed using the programme Origin7.0 from Micro Cal and fitted using the one-site binding model.

### Multi-angle light scattering

For MALS measurement, OPTN(1–119), OPTN(1–119) E50K mutant, Trx-tagged TBK1(677–729) or Trx-tagged TBK1(677–729) E696K mutant sample (100 μl at a concentration of 20 μM) was injected into an AKTA FPLC system with a Superose 12 10/300 GL column (GE Healthcare) with the column buffer containing 50 mM Tris-HCl, 100 mM NaCl, 1 mM DTT and 1 mM EDTA at pH 7.5. The chromatography system was coupled to a static light-scattering detector (miniDawn, Wyatt Technology) and a differential refractive index detector (Optilab, Wyatt Technology). Data were collected every 0.5 s with a flow rate of 0.5 ml min^−1^. Data were analysed using ASTRA 6 (Wyatt Technology, USA).

### Cell culture with transfection and fluorescence imaging

HeLa cells were cultured in Dulbecco's modified Eagle's medium (Invitrogen) supplemented with 10% fetal bovine serum (FBS, Invitrogen). Transfections of mCherry-OPTN, GFP-TBK1(kinase-dead) or related mutant plasmids were performed with Lipofectamine 2000 (Invitrogen) according to the manufacturer's instructions. After 24 h, cells were fixed with 4% paraformaldehyde and punched with 0.2% Triton X-100/PBS, and the nuclei were visualized by staining with 4,6-diamidino-2-phenylindole (DAPI). The cell images were captured and analysed using the TCS SP5 confocal microscope equipped with LAS X software (Leica, Inc., Thornwood, NY, USA). Particularly, the Pearson correlation coefficient analysis was performed using the LAS X software based on a randomly selected region that roughly contains one co-transfected HeLa cell. The statistical data represent mean±s.d. of >50 analysed cells (selected regions) from two independent experiments. The unpaired Student's *t*-test analysis was used to define a statistically significant difference.

### Autophagic degradation of GFP-htt140Q aggregates assay

HeLa cells were cultured in DMEM (Invitrogen) supplemented with 10% FBS (Invitrogen). Co-transfections of GFP-htt140Q with a control vector or Flag-OPTN variants were performed with Lipofectamine 2000 (Invitrogen) according to the manufacturer's instructions. After 48 h, cells were fixed with 4% paraformaldehyde and punched with 0.2% Triton X-100/PBS, and the nuclei were visualized by staining with DAPI. The cell images were captured using Olympus 1X81 microscope equipped with Micro Manger 1.4 software System. (Olympus Inc., Japan).

### Co-IP assay

For Co-IP, the Flag-tagged OPTN and GFP-tagged TBK1 fragments plasmids were transfected into HEK293T cells using Lipofectamine 2000 (Invitrogen), after 24 h transfection, the cells were lysed in the ice-cold cell lysis buffer (50 mM Tris (pH 7.5), 50 mM NaCl, 0.5% Nonidet P-40, 5% glycerol, 1 mM phenylmethylsulfonyl fluoride and 1% protease inhibitor cocktail) for 1 h at 4 °C, and followed by centrifugation at 12,000*g* for 15 min at 4 °C. The supernatant was then incubated with anti-Flag-conjugated agarose beads (Sigma, catalogue no. A2220) for 4 h at 4 °C. The beads were washed with the cell lysis buffer and re-suspended with SDS–PAGE sample buffer. The prepared samples were separated by 10% SDS–PAGE and analysed by western blot using anti-Flag (Sigma, catalogue no. F1804, 1:3,000 dilutions) and anti-GFP antibodies (Abmart, catalogue no. M20004M, 1:5,000 dilutions). The results are summarized in Supplementary Fig. 13.

### Data availability

The coordinates and structure factors of the OPTN(26–103)/TBK1 CTD complex, OPTN(26–103) E50K/TBK1 CTD mutant complex and NAP1 TBD/TBK1 CTD complex have been deposited in the Protein Data Bank under the accession codes 5EOF, 5EOA and 5EP6. All additional experimental data are available from the corresponding author on request.

## Additional information

**How to cite this article:** Li, F. *et al*. Structural insights into the interaction and disease mechanism of neurodegenerative disease-associated optineurin and TBK1 proteins. *Nat. Commun.* 7:12708 doi: 10.1038/ncomms12708 (2016).

## Supplementary Material

Supplementary Information Supplementary Figures 1-13 and Supplementary Tables 1-2.
