# Supplementary material for: Structural insights into the interaction and disease mechanism of neurodegenerative disease-associated optineurin and TBK1 proteins
Source: Nat Commun. 2016 Sep 13;7:12708. doi: 10.1038/ncomms12708 (PMC5027247; doi:10.1038/ncomms12708)
Supplement: Supplementary Information — Supplementary Figures 1-13 and Supplementary Tables 1-2. [file ncomms12708-s1.pdf]

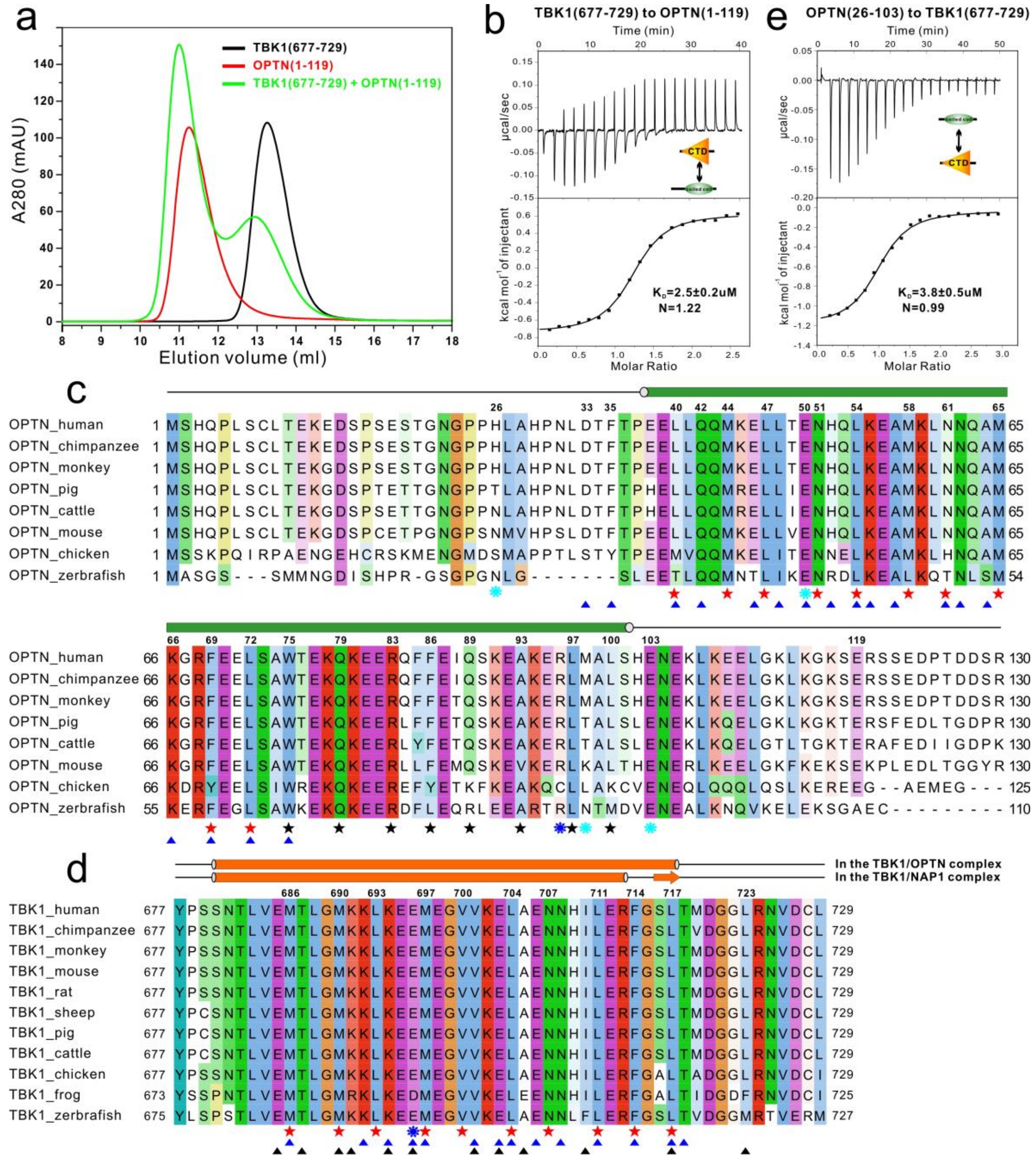

Supplementary Figure 1. Biochemical and sequence alignment analyses the interaction of OPTN and TBK1. (**a**) Analytical gel filtration chromatography analysis of the interaction between TBK1 CTD and OPTN(1-119). (**b**) The ITC-based measurement showing the binding affinity of TBK1 CTD with OPTN(1-119). The K_D error is the fitted error obtained from the data analysis software, when using the one-site binding model to fit the ITC data. The ITC-measured N value is related to the binding

stoichiometry. (**c**) Structure-based sequence alignment of N-terminal coiled-coil region of OPTN from different species. In this alignment, the conserved residues are highlighted by colors using software Jalview2.8.1 (http://www.jalview.org/). The residues that mediated the interior interactions, surface interactions and OPTN dimerization in the OPTN/TBK1 complex are highlighted with red stars, blue triangles and black stars, respectively. The four POAG-related residues and one ALS-related residue are indicated with cyan and blue gears, respectively. Specifically, the residues involved in the binding interfaces are further labeled with black numbers. (**d**) Structure-based sequence alignment of TBK1 CTD region from different species. In this alignment, the conserved residues are highlighted by colors using software Jalview2.8.1 (http://www.jalview.org/). The residues that are important for the interior interactions in OPTN/TBK1 and NAP1/TBK1 complexes are highlighted with red stars. The residues that are important for the surface interactions in OPTN/TBK1 and NAP1/TBK1 complexes are highlighted with blue triangles and black triangles, respectively. The ALS-associated mutation residue is further indicated with a blue gear. (**e**) The ITC-based measurement showing the binding affinity of TBK1 CTD with OPTN(26-103). The K_D error is the fitted error obtained from the data analysis software, when using the one-site binding model to fit the ITC data. The ITC-measured N value is related to the binding stoichiometry.

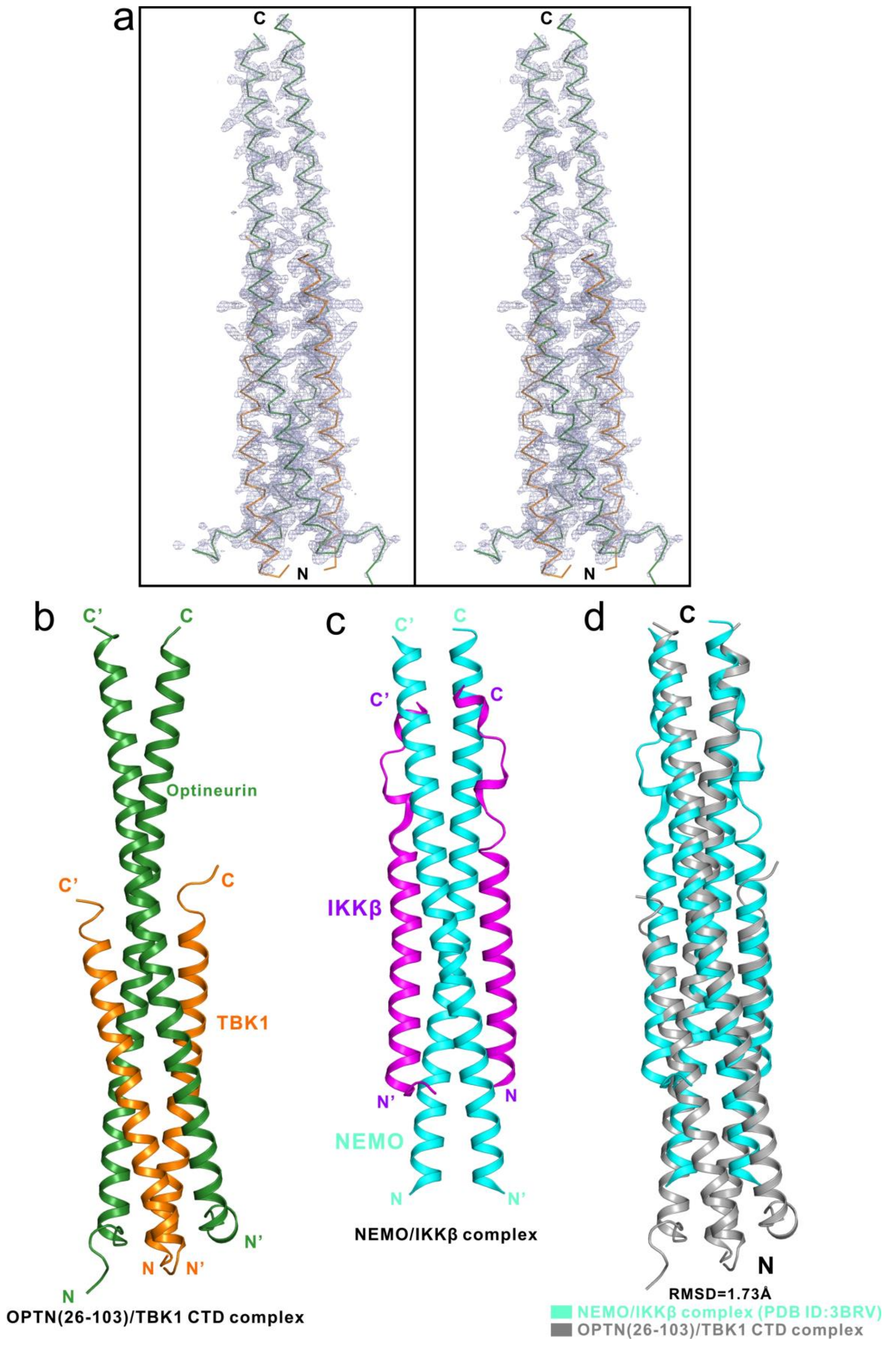
a
C
N
C
N
b
C'
C
Optineurin
C'
C
TBK1
N'
N
N'
N
OPTN(26-103)/TBK1 CTD complex
c
C'
C
C'
C
IKKβ
N'
N
NEMO
N
N'
NEMO/IKKβ complex
d
C
N
RMSD=1.73Å
NEMO/IKKβ complex (PDB ID:3BRV)
OPTN(26-103)/TBK1 CTD complex

Supplementary Figure 2. Structural comparison of the OPTN/TBK1 complex with the NEMO/IKKβ complex. (**a**) $2F_{observed}$-$F_{calculated}$ electron density map for the OPTN(26-103)/TBK1 CTD complex structure. The map is contoured at 2.0σ, and only the main-chains of proteins are shown in the ribbon mode for simplicity. (**b**) Ribbon diagram showing the overall structure of OPTN(26-103)/TBK1 CTD complex. In this drawing, the OPTN(26-103) is shown in forest green, and TBK1 CTD in orange. (**c**) Ribbon diagram showing the overall structure of NEMO/IKKβ complex. In this drawing, the IKKβ is shown in magenta, and NEMO in cyan. (**d**) The ribbon diagram showing the structural comparison of the OPTN/TBK1 complex (grey) and the NEMO/IKKβ complex (cyan).

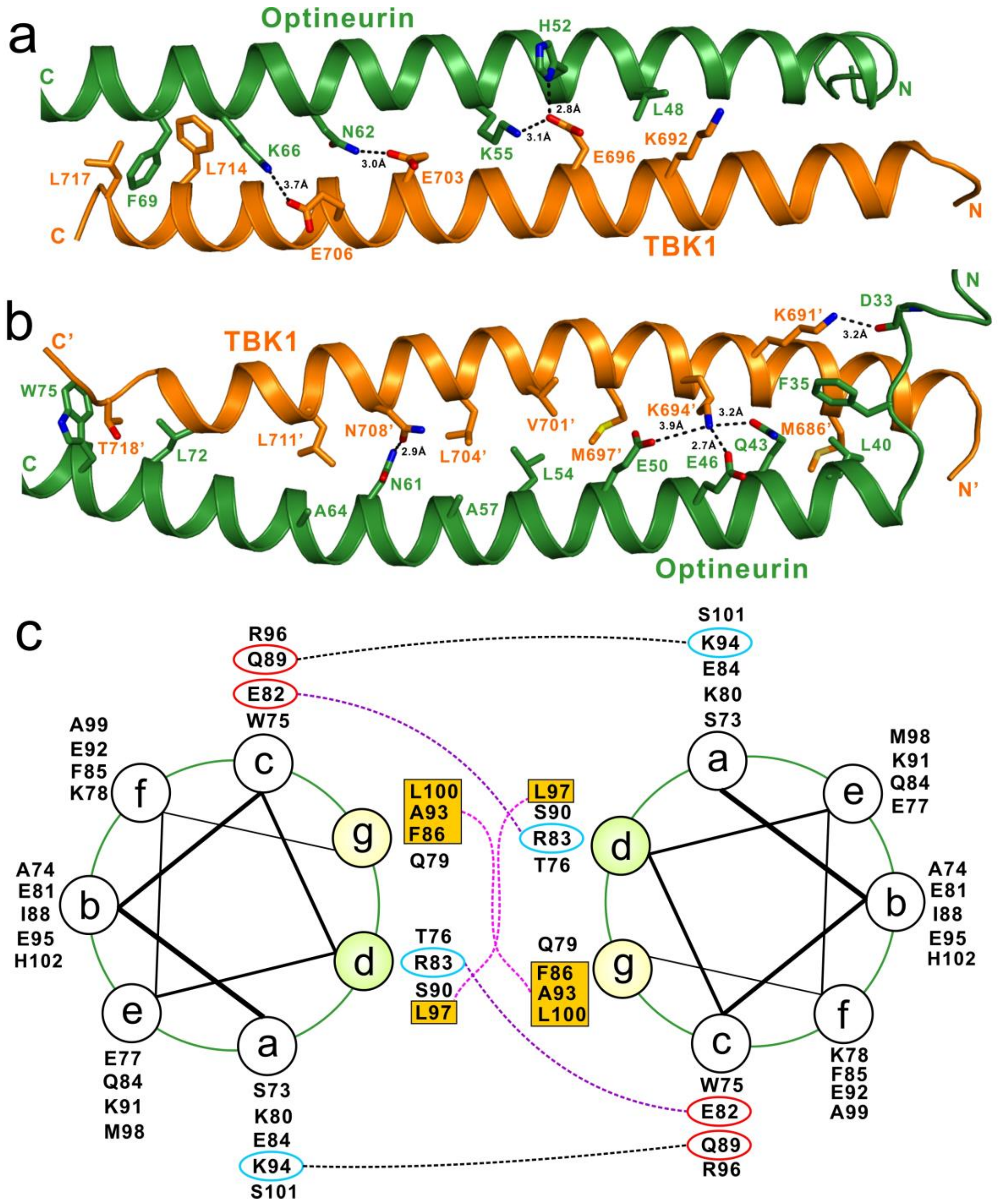

Supplementary Figure 3. Detailed surface interactions in the OPTN(26-103)/TBK1 CTD complex structure. (**a** and **b**) The combined ribbon representation and stick model showing the detailed two asymmetrical surface interactions between OPTN(26-103) and TBK1 CTD in the OPTN(26-103)/TBK1 CTD complex. In this drawing, the OPTN(26-103) is shown in forest green, and the TBK1 CTD in orange. The residues that are

important for surface interactions are shown in stick model and labeled with numbers. The related salt bridges and hydrogen bonds are indicated by black dash lines. (**c**) Helical wheel presentation showing the detailed dimerization interface formed between two 4 heptad repeats located at the C-terminal part of OPTN(26-103). In this presentation, the inter-helical hydrogen bonds, salt bridges and hydrophobic contacts are depicted by black, purple and pink dashed lines, respectively. Hydrophobic residues located at the "d" and "g" positions form the hydrophobic core of the OPTN coiled-coil dimer, and are further highlighted with orange boxes.

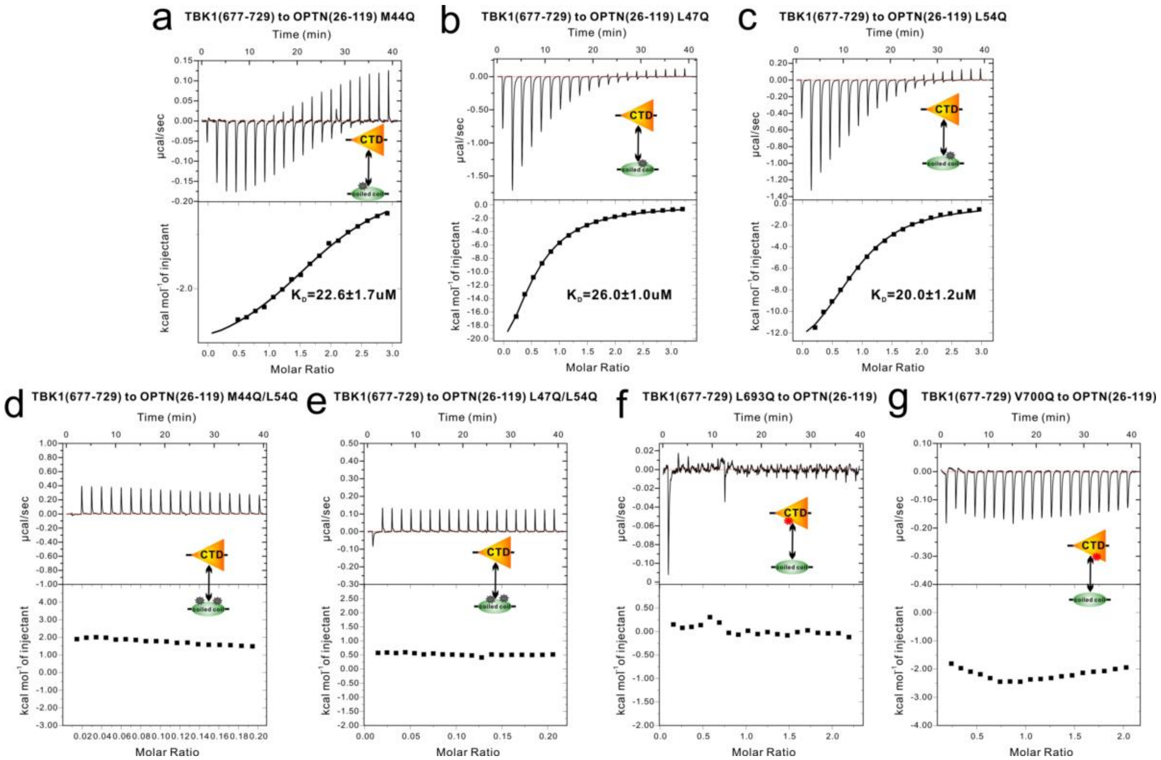

Supplementary Figure 4. Validations of the OPTN/TBK1 complex structure by mutations of key interface residues. (**a-e**) ITC-based measurements of the binding affinities of TBK1 CTD with OPTN(26-119) M44Q mutant (**a**), L47Q mutant (**b**), L54Q mutant (**c**), M44Q/L54Q mutant (**d**), and L47Q/L54Q mutant (**e**). (**f** and **g**) ITC-based

measurements of the binding affinities of OPTN(26-119) with TBK1 CTD L693Q mutant (**f**), and V700Q mutant (**g**). The K_D errors are the fitted errors obtained from the data analysis software, when using the one-site binding model to fit the ITC data. All these data confirm the specific interaction between OPTN(26-103) and TBK1 CTD observed in the complex structure.

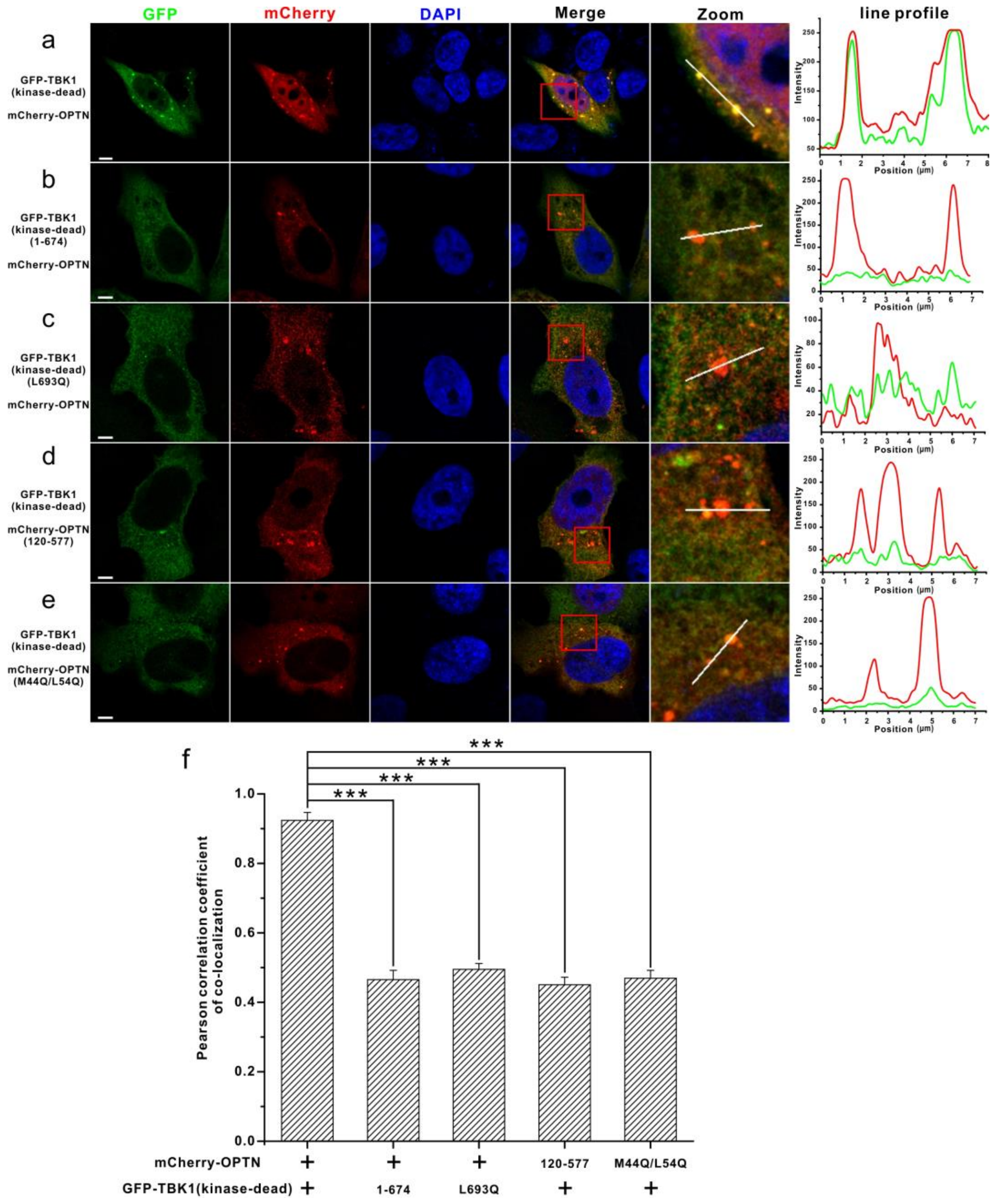

Supplementary Figure 5. The specific interaction between OPTN N-terminal coiled-coil domain and TBK1 CTD is required for cellular co-localizations of OPTN and kinase-dead TBK1 in transfected HeLa cells. (**a**) When co-transfected, the kinase-dead TBK1 co-localizes very well with the OPTN clusters in cytosol. Deletion of the TBK1 CTD (**b**) or the OPTN N-terminal coiled-coil region (**d**) largely abolishes the co-localization of TBK1 with OPTN. The TBK1 L693Q (**c**) and OPTN M44Q/L54Q (**e**) mutations that abrogate the TBK1/OPTN interaction *in vitro*, essentially eliminate the co-localization of kinase-dead TBK1 and OPTN. Since overexpression of wild type TBK1 in transfected HeLa cells dramatically induces cell death with unknown reasons, thereby, all the related TBK1 mutants used in the co-localization experiments were derived from the kinase-dead TBK1 S172A mutant. (**f**) Statistical result related to the co-localizations of OPTN and the kinase-dead TBK1 as well as their mutants in the co-transfected HeLa cells shown as Pearson correlation. The Pearson correlation coefficient analysis was performed using the LAS X software based on a randomly selected region that roughly contains one co-transfected HeLa cell. The data represent mean±s.d. of >50 analyzed cells (selected regions) from two independent experiments, and scale bar, 5 μm. The unpaired Student t-test analysis was used to define a statistically significant difference, and the stars indicate the significant differences between the indicated bars (***P<0.001).

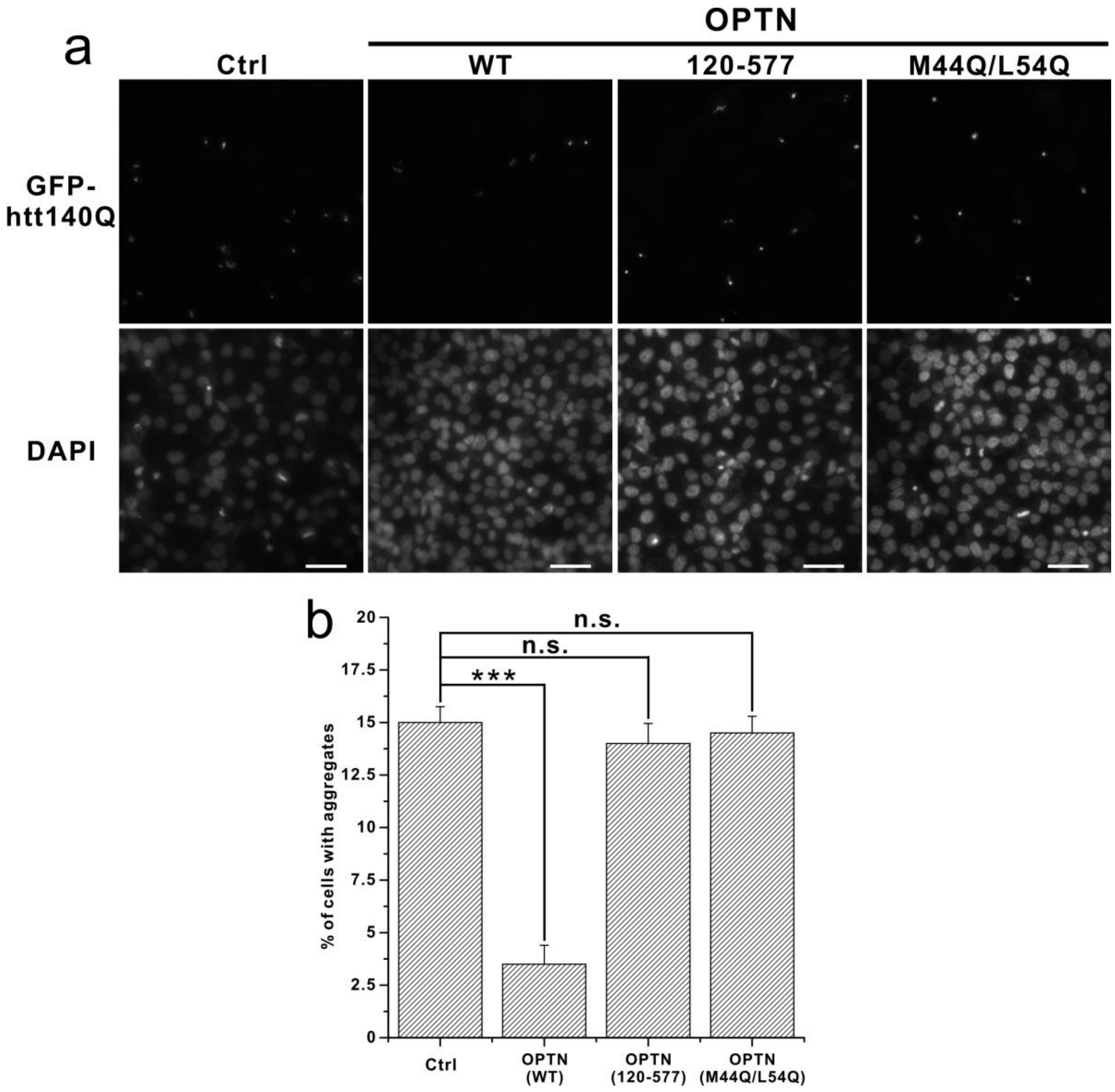

Supplementary Figure 6. The specific interaction between OPTN N-terminal coiled-coil domain and TBK1 CTD was necessary for the OPTN-mediated autophagic degradation of GFP-htt140Q aggregates in transfected HeLa cells. (**a**) Representative images of HeLa cells co-expressing GFP-htt140Q and different OPTN variants acquired by the Olympus 1X81 microscope equipped with Micro Manger 1.4 software System. In this assay, the GFP-htt140Q was co-transfected with control vector, wild type OPTN, truncation mutant OPTN(120-577), or TBK1-binding-defective OPTN(M44Q/L54Q) mutant in HeLa cells, after 48 hours, the cell images containing GFP-positive puncta were captured, and the nucleus were shown by staining with DAPI. Scale bar, 50 µm. (**b**) Quantitative measurements of GFP-positive aggregates in the cell images acquired in (**a**).

All quantified data represent mean±s.d. of results collected from 3 independently performed experiments. The unpaired Student t-test analysis was used to define a statistically significant difference, and the stars indicate the significant differences between the indicated bars (***P<0.001) and n.s. stands for not significant.

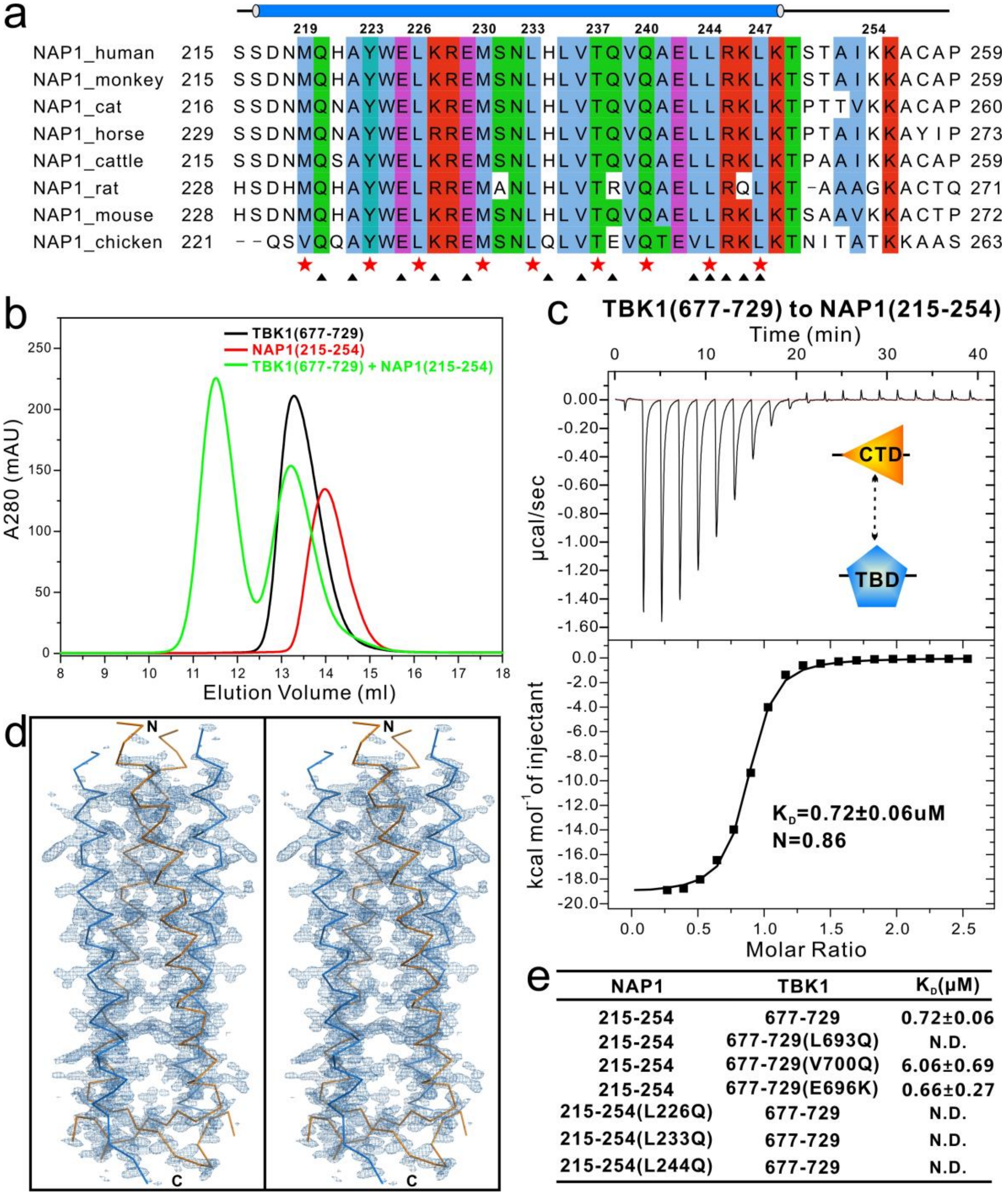

NAP1	TBK1	K_D(μM)
215-254	677-729	0.72±0.06
215-254	677-729(L693Q)	N.D.
215-254	677-729(V700Q)	6.06±0.69
215-254	677-729(E696K)	0.66±0.27
215-254(L226Q)	677-729	N.D.
215-254(L233Q)	677-729	N.D.
215-254(L244Q)	677-729	N.D.

Supplementary Figure 7. Biochemical characterizations of the interaction between

TBK1 and NAP1. (**a**) Structure-based sequence alignment of TBK1-binding region of NAP1 from different species. In this alignment, the conserved residues are highlighted by colors using software Jalview2.8.1 (http://www.jalview.org/). The residues that mediated the interior interactions and surface interactions in the NAP1 TBD/TBK1 CTD complex are highlighted with red stars and black triangles, respectively. Specifically, the residues involved in the interior interactions are also labeled with black numbers. (**b**) Analytical gel filtration chromatography analysis of the interaction between TBK1 CTD and NAP1 TBD. (**c**) ITC-based measurement showing the binding affinity of TBK1 CTD with NAP1 TBD. The K_D error is the fitted error obtained from the data analysis software, when using the one-site binding model to fit the ITC data. The ITC-measured N value is related to the binding stoichiometry. (**d**) $2F_{observed}$-$F_{calculated}$ electron density map for the NAP1 TBD/TBK1 CTD complex structure. The map is contoured at 2.0σ, and only the main-chains of proteins are shown in the ribbon mode for simplicity. (**e**) A statistic table summarizing the measured binding affinities between NAP1 TBD and TBK1 CTD protein or their mutants by ITC-based binding assay. ‘N.D.’ stands for that the K_D value is not detectable. The K_D errors are the fitted errors obtained from the data analysis software, when using the one-site binding model to fit the ITC data.

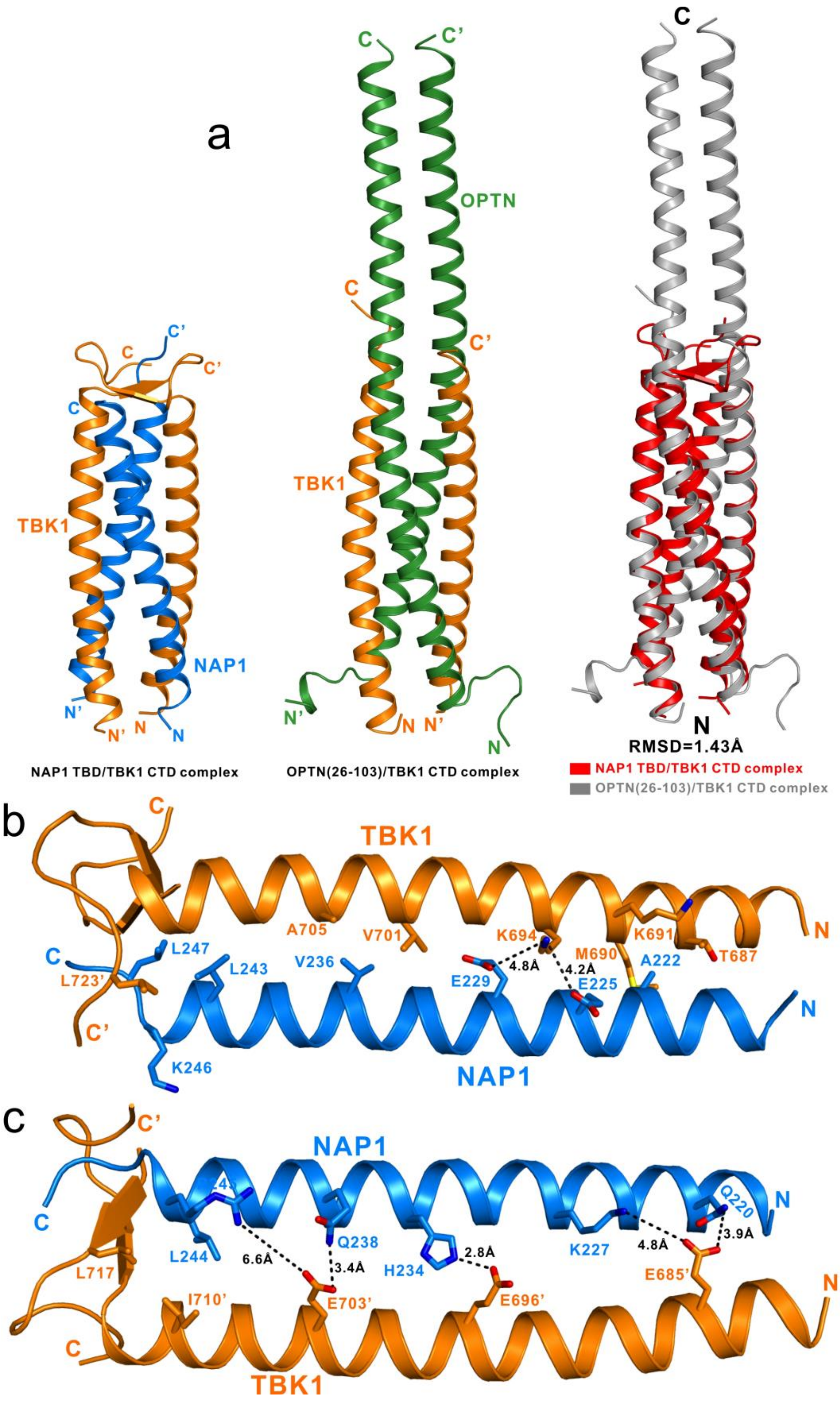
a
c
NAP1 TBD/TBK1 CTD complex
OPTN(26-103)/TBK1 CTD complex
RMSD=1.43Å
NAP1 TBD/TBK1 CTD complex
OPTN(26-103)/TBK1 CTD complex
TBK1
NAP1
OPTN
b
TBK1
NAP1
A705
V701
K694
M690
K691
T687
L247
L243
V236
E229
E225
A222
L723'
K246
4.8Å
4.2Å
c
NAP1
TBK1
Q220
L244
Q238
H234
K227
L717
I710'
E703'
E696'
E685'
6.6Å
3.4Å
2.8Å
4.8Å
3.9Å

Supplementary Figure 8. Detailed structural analysis of the NAP1 TBD/TBK1 CTD complex. (**a**) Structural comparison of the NAP1 TBD/TBK1 CTD complex with the OPTN(26-103)/TBK1 CTD complex. In this diagram, the left panel showing the overall structure of NAP1 TBD/TBK1 CTD complex, the middle panel showing the overall structure of OPTN(26-103)/TBK1 CTD complex, and the right panel showing the superposed structures of NAP1 TBD/TBK1 CTD complex (red) and OPTN(26-103)/TBK1 CTD complex (gray). (**b** and **c**) The combined ribbon representation and stick model showing the detailed two asymmetrical surface interactions between NAP1 TBD and TBK1 CTD in the NAP1 TBD/TBK1 CTD complex. In these drawings, the residues that are important for surface interactions are labeled with numbers, and their side chains are shown in stick model. The involved hydrogen bonds, salt bridges and charge-charge interactions are indicated by black dash lines.

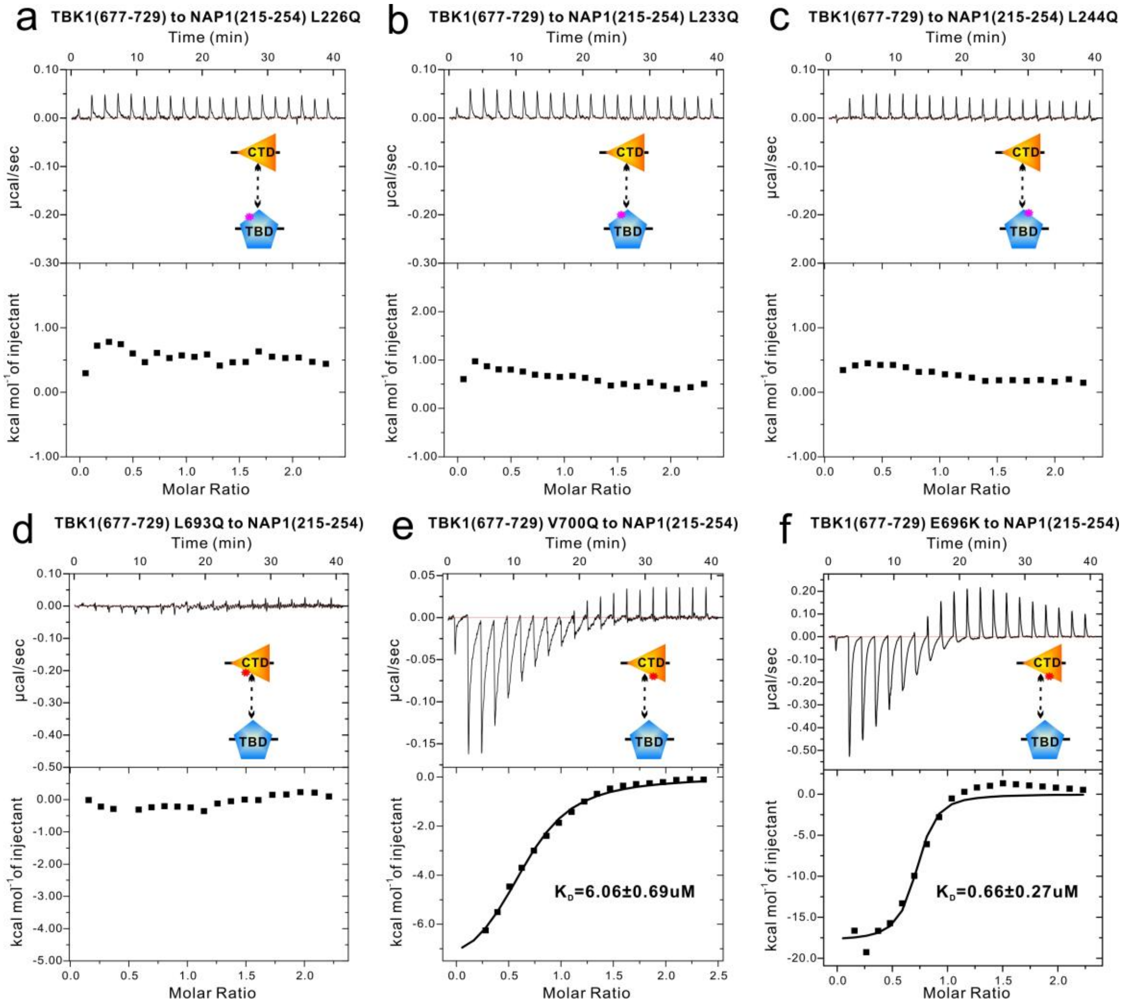

Supplementary Figure 9. Validations of the NAP1 TBD/TBK1 CTD complex structure by mutations of key interface residues. (**a**-**c**) ITC-based measurements of the binding affinities of TBK1 CTD with NAP1 TBD L226Q mutant (**a**), L233Q mutant (**b**), and L244Q mutant (**c**). (**d**-**f**) ITC-based measurements of the binding affinities of NAP1 TBD with TBK1 CTD L693Q mutant (**d**), V700Q mutant (**e**), and E696K mutant (**f**). The K_D errors are the fitted errors obtained from the data analysis software, when using the one-site binding model to fit the ITC data. All these data confirm the specific interactions between NAP1 TBD and TBK1 CTD observed in the complex structure.

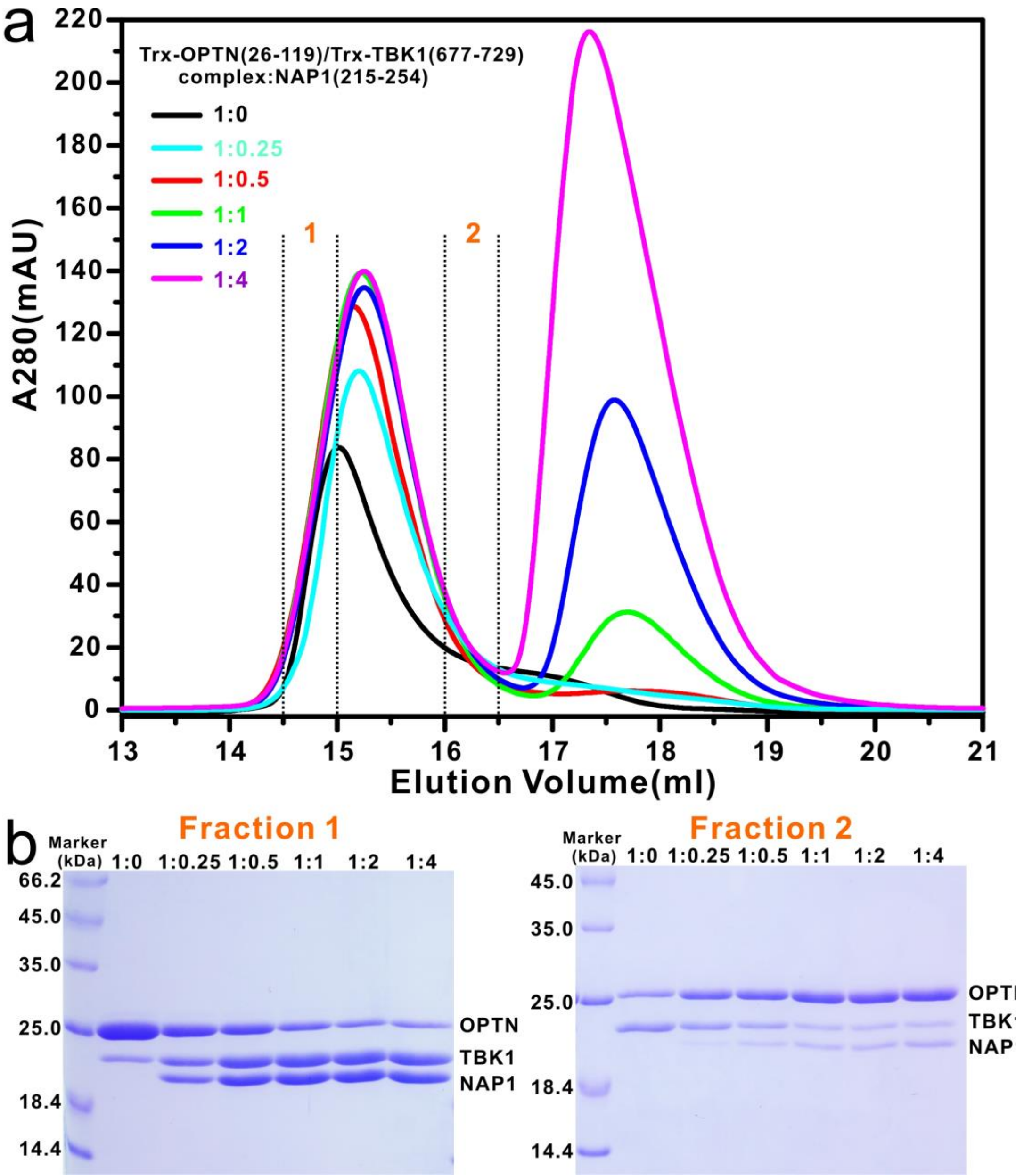

Supplementary Figure 10. OPTN and NAP1 are competitive in binding to TBK1. (**a**) Analytic gel filtration chromatography analyses of the OPTN(26-119)/TBK1(677-729) complex incubated with increasing molar ratio of NAP1(215-254) proteins. (**b**) SDS-PAGE combined with Coomassie-blue staining analyses showing the protein components of corresponding fraction1 and fraction2 collected from different analytic gel filtration chromatography experiments in panel A.

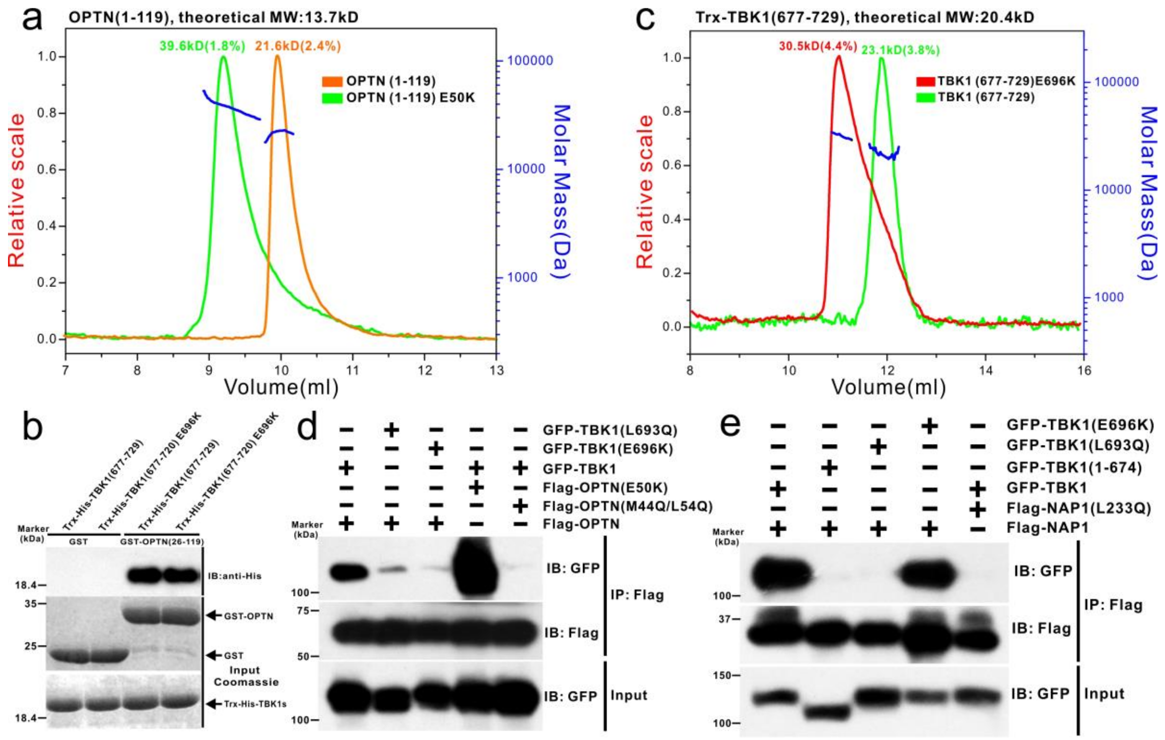

Supplementary Figure 11. Biochemical analyses of the effects induced by disease-associated OPTN E50K and TBK1 E696K mutations. (**a**) Multi-angle light scattering (MALS) analyses of wild type and E50K mutant of OPTN(1–119) proteins, showing the relative light scattering signals as a function of elution volume. The derived molecular masses of wild type and E50K mutant of OPTN(1–119) are shown in orange and green, respectively. The molecular masses errors were the fitted errors obtained from the data analysis software and included in the brackets. (**b**) GST pull-down analysis of the interaction between OPTN(26-119) and TBK1 CTD E696K mutant, showing that the TBK1 E696K mutation does not abolish the interaction between OPTN(26-119) and TBK1 CTD. (**c**) MALS analyses of wild type and E696K mutant of TBK1(677–729) proteins, showing the relative light scattering signals as a function of elution volume. The gel filtration peak profiles and derived molecular masses of wild type and E696K mutant of OPTN(677–729) are shown in green and red, respectively. (**d**) Co-

immunoprecipitation assay showing that the full length OPTN interacts well with TBK1 in transfected HEK293T cells, while the structural-based OPTN M44Q/L54Q mutation and TBK1 L693Q mutation as well as the ALS-associated TBK1 E696K mutation essentially abolish the specific interaction between OPTN and TBK1 in transfected cells, and the POAG-related OPTN E50K mutation largely enhances the interaction of OPTN with TBK1. (**e**) Co-immunoprecipitation assay showing that the full length NAP1 interacts well with TBK1 in transfected cells, and the structural-based NAP1 L233Q mutation and TBK1 L693Q mutation as well as the TBK1 truncation mutation deleting its CTD domain (TBK1(1-674)) completely abolish the specific interaction between OPTN and TBK1 in cells, while the ALS-associated TBK1 E696K mutation has little effect on the interaction between OPTN and TBK1 in cells.

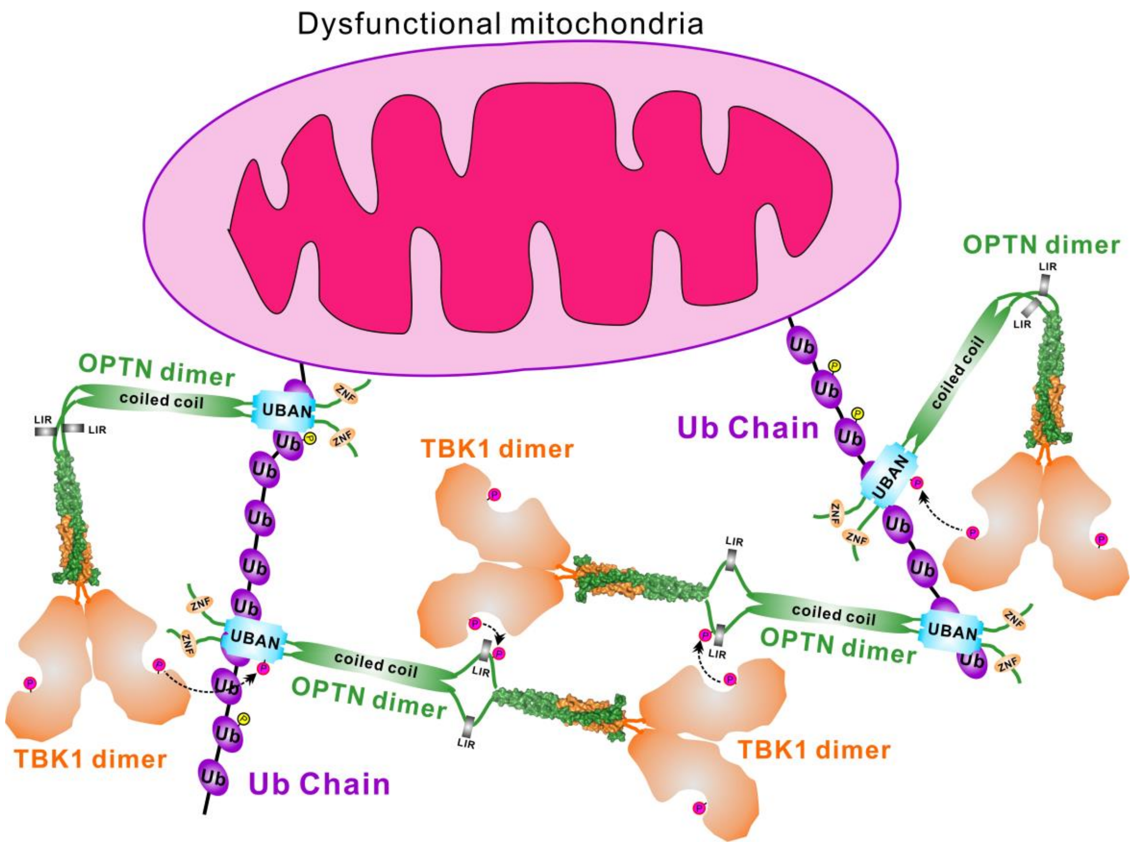

Supplementary Figure 12. A proposed cartoon model illustrating the recruitment of OPTN and TBK1 complex to ubiquitin-decorated dysfunctional mitochondria as well as the regulation of OPTN by TBK1-mediated phosphorylation in mitophagy.

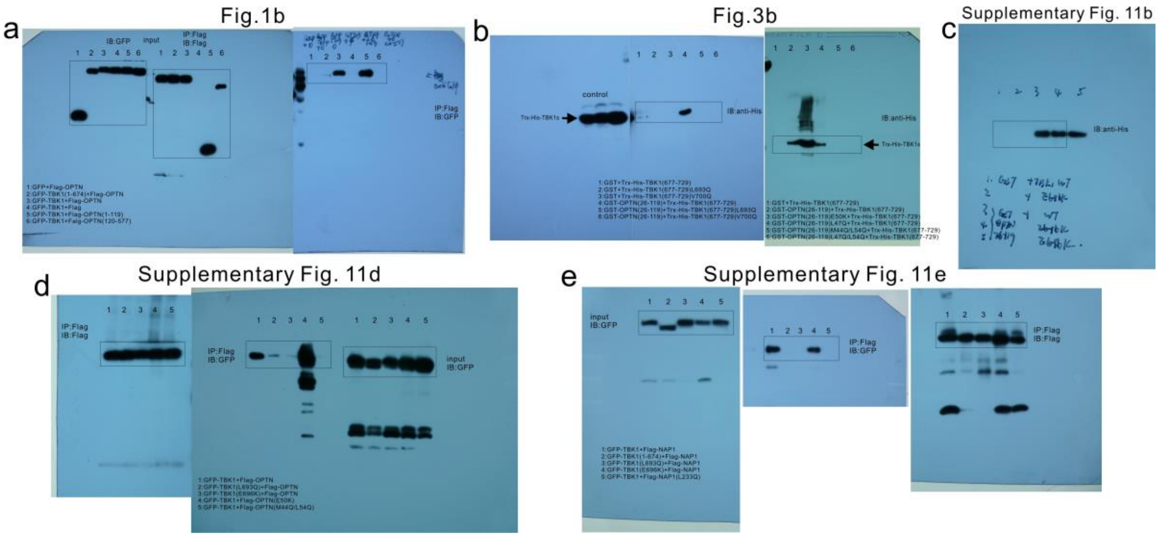

Supplementary Figure 13. The uncropped figures of pull-down and Co-IP experiments. (a-e) The original western blots pictures of Fig.1b **(a),** Fig.3b **(b),** supplementary Fig.11b **(c),** supplementary Fig.11d (**d**), and supplementary Fig.11e (**e**), respectively.

Supplementary Table 1
Statistics of X-ray crystallographic data collection and model refinements

	SeMet-OPTN(26-119)/TBK1(677-729)	OPTN(26-103)/TBK1(677-729)
**Data collection**		
Wavelength (Å)	0.9791	0.9785
Space group	P 21 21 21	P 21 21 21
Cell dimensions		
a, b, c (Å)	42.076, 51.678, 144.69	36.321, 54.775, 153.141
α, β, γ (^{o})	90, 90, 90	90, 90, 90
Resolution range (Å)	50.00 - 2.50 (2.54 - 2.50)	50.00 - 2.05 (2.09 - 2.05)
R_{merge} (%)[a]	11.7 (85.3)	5.2 (63.5)
I / σI	32.98 (4.51)	14.85 (2.53)
Completeness (%)	99.17 (95.72)	99.9 (99.65)
Redundancy	11.7 (11.5)	6.4 (6.4)
**Refinement**		
Resolution (Å)		50.00 - 2.05 (2.09 - 2.05)
No. reflections		19973
R_{work} / R_{free} (%)[b]		20.60 (27.48) / 25.49 (29.05)
No. of atoms		
Protein		1916
Ligand		0
Water		125
Average B-factor ($Å^2$)		36.9
R.m.s.deviations		
Bond lengths (Å)		0.007
Bond angles (°)		0.93
Ramachandran plot[c]		
Favored region (%)		97.0
Allowed region (%)		3.0
Outliers (%)		0

[a] $R_{merge} = \sum|I_i - I_m|/\sum I_i$, where I_i is the intensity of the measured reflection and I_m is the mean intensity of all symmetry related reflections.

[b] $R_{work} = \Sigma||F_{obs}| - |F_{calc}||/\Sigma|F_{obs}|$, where F_{obs} and F_{calc} are observed and calculated structure factors.

$R_{free} = \Sigma_T||F_{obs}| - |F_{calc}||/\Sigma_T|F_{obs}|$, where T is a test data set of about 5% of the total reflections randomly chosen and set aside prior to refinement.

[c] Defined by Molprobity.

Numbers in parentheses represent the value for the highest resolution shell.

Supplementary Table 2

Statistics of X-ray crystallographic data collection and model refinements

	OPTN(26-103) E50K/TBK1(677-729)	NAP1(215-254)/TBK1(677-729)
**Data collection**		
Wavelength (Å)	0.9786	0.9793
Space group	P 21 21 21	P 21 21 21
Cell dimensions		
a, b, c (Å)	36.824, 54.549, 153.305	41.36, 50.113, 84.416
α, β, γ (^{o})	90, 90, 90	90, 90, 90
Resolution range (Å)	50.00 - 2.50 (2.54 - 2.50)	50.00 - 1.45 (1.48 - 1.45)
R_{merge} (%)[a]	6.9 (41.4)	6.0 (52.3)
I / σI	10.24 (2.81)	27.50 (4.67)
Completeness (%)	99.72(98.36)	98.77 (90.68)
Redundancy	3.5 (3.3)	6.8 (5.7)
**Refinement**		
Resolution (Å)	50.00 - 2.50 (2.54 - 2.50)	50.00 - 1.45 (1.48 - 1.45)
No. reflections	11206	31329
R_{work} / R_{free} (%)[b]	19.89 (26.74) / 26.77 (37.26)	18.02 (20.39) / 20.82 (24.17)
No. of atoms	11206	31329
Protein	1889	1471
Ligand	0	30
Water	24	112
Average B-factor (Å^2)	58.3	31.4
R.m.s.deviations		
Bond lengths (Å)	0.007	0.006
Bond angles (°)	0.9	1.05
Ramachandran plot[c]		
Favored region (%)	99.0	99.0
Allowed region (%)	1.0	1.0
Outliers (%)	0	0

[a] $R_{merge} = \sum|I_i - I_m|/\sum I_i$, where I_i is the intensity of the measured reflection and I_m is the mean intensity of all symmetry related reflections.

[b] $R_{work} = \Sigma||F_{obs}| - |F_{calc}||/\Sigma|F_{obs}|$, where F_{obs} and F_{calc} are observed and calculated structure factors.

$R_{free} = \Sigma_T||F_{obs}| - |F_{calc}||/\Sigma_T|F_{obs}|$, where T is a test data set of about 5% of the total reflections randomly chosen and set aside prior to refinement.

[c] Defined by Molprobity.

Numbers in parentheses represent the value for the highest resolution shell.
